# Disrupting SRSF10-dependent BCAT2 exon skipping reprograms tumor-associated macrophages and enhances anti-PD-1 efficacy in gastric cancer

**DOI:** 10.1038/s41419-026-08622-3

**Published:** 2026-04-22

**Authors:** Xiao-Bo Huang, Xin-Peng Yang, Hua-Long Zheng, Ling-Qian Wang, Chen-Yang Jiang, Yun-Lin Chen, Bin Lin, Yi-Fan Li, Xiao-Jing Guo, Qiang Huang, You-Xin Gao, Yi Li, Xiao-Qian Ye, Jia-Bin Wang, Jian-Wei Xie, Jian-Xian Lin, Chao-Hui Zheng, Chang-Ming Huang, Qi-Yue Chen, Ping Li

**Affiliations:** 1 Department of Gastric Surgery, Fujian Medical University Union Hospital, Fuzhou, Fujian PR China; 2 Key Laboratory of Ministry of Education of Gastrointestinal Cancer, Fujian Medical University, Fuzhou, Fujian PR China; 3 Fujian Key Laboratory of Tumor Microbiology, Fujian Medical University, Fuzhou, Fujian PR China

**Keywords:** Gastric cancer, Cancer microenvironment

## Abstract

Aberrant alternative splicing (AS) in cancer generates oncogenic proteomic diversity that drives tumor progression. Given the suboptimal efficacy of immune checkpoint inhibitors (ICIs) in gastric cancer (GC), the therapeutic potential of modulating RNA splicing to augment immunotherapy remains unclear. Here, we demonstrate that the splicing factor SRSF10 is progressively upregulated during gastric tumorigenesis and exhibits elevated expression in ICIs-resistant GC. Utilizing multiple mouse models, we confirmed that SRSF10 ablation with a selective inhibitor 1C8 robustly inhibits GC growth and enhances CD8^+^ T-cell infiltration via CCL2-mediated reprogramming of tumor-associated macrophages (TAMs). Notably, SRSF10 blockade restricts pre-neoplastic metaplastic cells re-entry the cell cycle and the TAMs reprogramming. Mechanistically, cell-autonomous SRSF10 activates mTOR signaling primarily through inclusion of exon 2 in the BCAA transaminase 2 (BCAT2) mRNA. Pharmacological antagonism of SRSF10 potentiated the therapeutic effect of anti-PD-1 antibody in *Tff1-CreER*^*T2*^*; Apc*^*fl/fl*^*; p53*^*fl/fl*^ orthotopic GC models. Collectively, our findings revealed that SRSF10 orchestrates mTOR-CCL2 signaling by alternative RNA splicing of BCAT2 to reprogram TAMs, proposing SRSF10 as a tempting therapeutic target for GC immunotherapy.

## Introduction

Immune checkpoint inhibitors (ICIs) have revolutionized cancer therapeutics, emerging as one of the most promising therapeutic strategies [1, 2]. Despite ~30% of solid tumor patients achieve durable antitumor responses to ICIs treatment, clinical efficacy remains constrained by primary resistance or acquired resistance following initial response [1–4]. Compounding this challenge, gastric cancer (GC) manifests a profoundly heterogeneous malignancy characterized by substantial variability in its responsiveness to immunotherapy [5–9]. This variability underscores the urgent need to decipher the complex molecular underpinnings of ICIs refractoriness.

The immunosuppressive tumor microenvironment (TME), characterized by abundant tumor-associated macrophages (TAMs), constitutes a major impediment to ICIs efficacy in malignancies including GC [10, 11]. As pivotal components, TAMs encompass two functionally distinct subsets: immunosuppressive M2-polarized macrophages and pro-inflammatory M1 macrophages [12–14]. M2 macrophages perpetuate immunosuppression by secreting cytokines such as IL-10 and TGF-β, thereby dampening immune cell activity [12–14]. Conversely, M1 macrophages potentiate T-cell-mediated anti-tumor responses via molecules including CXCL9 and NOS2, facilitating tumor cell eradication [12–14]. Crucially, the inherent plasticity of macrophages presents a therapeutic opportunity to reprogram these cells toward a pro-inflammatory, antitumoral M1-like state, transforming immunologically “cold” tumors into “hot” tumors and enhancing ICIs responsiveness in GC [11, 14].

With advancing comprehension of cancer molecular pathogenesis, there is increasing recognition of the critical function transcriptional reprogramming in both tumor progression and immune evasion [15]. Notably, we emphasize the discovery of a clinically relevant gene signature associated with immunosuppressive therapy resistance, which holds potential for elucidating the dynamic mechanisms underlying immunotherapy resistance in GC: alternative splicing (AS) [16–19]. AS is a nuclear process involving intron excision from pre-mRNA and exon ligation to generate mature RNAs [16–19]. Previous studies revealed that >90% of human multi-exon genes undergo AS, establishing it as a critical post-transcriptional mechanism governing gene expression and cellular fate [17–20]. The serine/arginine-rich (SR) family members act as auxiliary splicing regulators by binding exonic/intronic splicing enhancers (ESEs/ISEs) or silencers (ESSs/ISSs) near splice sites [19, 21, 22]. Although SR proteins are evolutionarily conserved, their dysregulation or mutation in cancer drives tumorigenesis by altering pre-mRNA splicing, metabolism, decay, and translation [22, 23]. SRSF10, a member of SR family, is frequently overexpressed in human malignancies and promotes oncogenic transformation, with its biological implications increasingly explored [24, 25]. However, the role of SRSF10 in gastric tumorigenesis and relevance to immunotherapy remain elusive.

Here, by employing conditional transgenic mouse models and extensive human samples, we unveil that elevated SRSF10 correlates with diminished responsiveness to ICIs in GC, exhibiting progressive elevation concomitant with each stage of tumorigenesis. Mechanistically, ablation of SRSF10 potentiates macrophage reprogramming via reduced CCL2 secretion, thereby suppressing neoplastic proliferation. Intriguingly, we demonstrate that SRSF10 orchestrates mTOR signaling activation during tumorigenesis and establishment of the immunosuppressive niche, mediated through regulation of BCAT2 exon 2 alternative RNA splicing. Finally, we propose targeting SRSF10 synergized with anti-PD-1 by reshaping macrophage plasticity, proposing a druggable strategy to overcome GC immunotherapy resistance.

## Methods

### Mice

All animal studies and procedures were conducted in accordance with the guidelines of the Animal Protection Committee of Fujian Medical University and were approved by the Ethics Committee of Fujian Medical University/Laboratory Animal Center (No. FJMU IACUC 2021-J-0174). *Apc*^*fl/fl*^ (Cat# 029275), *p53*^*fl/fl*^ (Cat# 008462) and *Rosa26-LSL-Tdtomato* (Cat# 007914) mice were purchased from the Jackson Laboratory (Bar Harbor, Maine, USA). *Tff1-CreER*^*T*^^*2*^ mice were purchased from the Shanghai Model Organisms Center (Shanghai, China). C57BL/6 wild-type mice were purchased from Cyagen Biosciences (Suzhou, China). All mice used were 6–8 weeks of age. Mice were given intraperitoneal injections of tamoxifen (T832955; MACKLIN, Shanghai, China) mixed with sunflower oil at the times shown in the text and/or figures. Samples were analyzed at the indicated time points.

### Statistical analysis

All statistical analyses in this study were performed using GraphPad Prism (version 10.2.1, San Diego, California, USA) or SPSS (version 19.0). Quantitative data are presented as the mean ± standard deviation (SD). Comparisons between two groups of variables were conducted using two-tailed Student’s *t* tests or non-parametric Mann–Whitney U-tests. Patient prognosis was assessed using Kaplan–Meier analysis. Log-rank tests were used to compare differences in survival rates between groups. Univariate and multivariate Cox proportional hazards models were employed to analyze prognostic parameters influencing overall survival (OS), and forest plots were constructed for evaluation. Spearman’s correlation analysis was used to assess the relationship between two variables. The chi-squared test was used to investigate the relationship between two categorical variables. Significance levels are indicated as follows: *P < 0.05, **P < 0.01, and ***P < 0.001.

Additional methods are detailed in the online supplementary materials.

## Results

### SRSF10 orchestrates ICIs resistance and gastric tumorigenesis

**Fig. 1:**
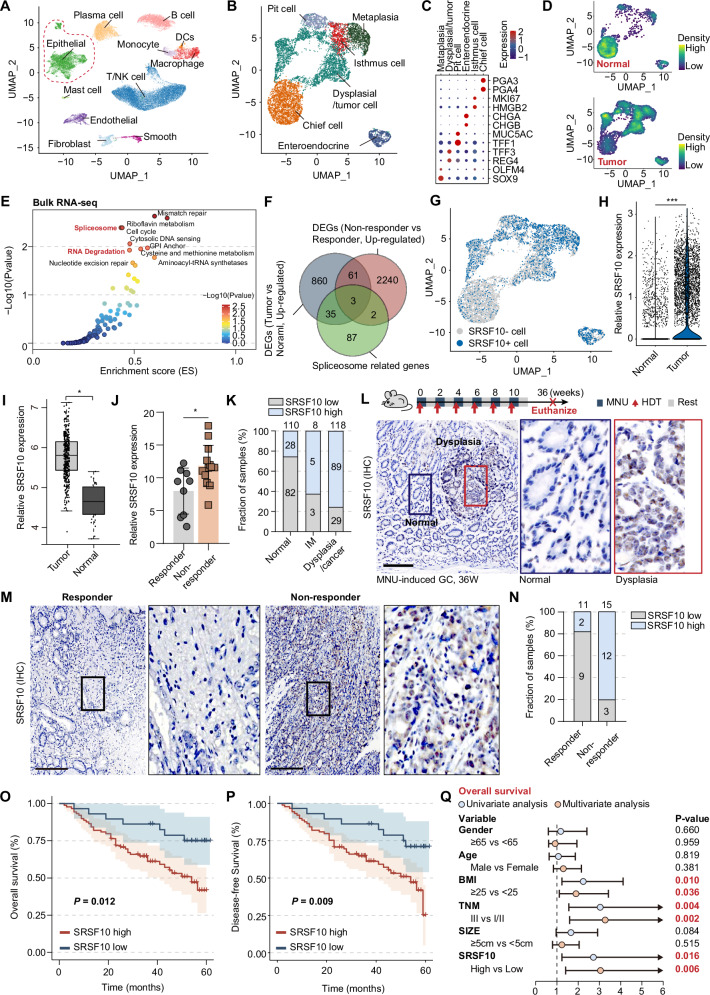
SRSF10 orchestrates ICIs resistance and gastric tumorigenesis. **A** UMAP visualization of gastric single-cell transcriptomes showing major cell populations. **B** Subclustering of epithelial cells identifies six distinct subsets. **C** Dot plot showing representative marker genes used to define epithelial subsets. **D** Density plots of epithelial cells in normal and tumor samples. **E** Bulk RNA-seq gene set enrichment analysis (GSEA) comparing non-responders versus responders to immune checkpoint inhibitors (ICIs), showing enrichment of spliceosome and RNA processing pathways. **F** Venn diagram of upregulated genes from scRNA-seq (Tumor vs. Normal), bulk RNA-seq (Non-responder vs. Responder), and spliceosome-related gene sets, identifying three overlapping candidates: *SRSF10*, *SRSF6*, and *THOC2*. **G** UMAP plot showing distribution of SRSF10-positive cells (blue) and SRSF10-negative cells (gray), with enrichment of SRSF10-positive cells in tumor populations. **H** Violin plot showing *SRSF10* expression in tumor compared with normal tissues. **I** The relative SRSF10 mRNA expression levels of SRSF10 in tumor and normal tissues in TCGA-STAD. **J** The relative SRSF10 expression in responder and non-responder Groups. **K** The bar chart showing the fraction of samples with low or high SRSF10 expression across different tissue types (normal, intestinal metaplasia, dysplasia/cancer). **L** A schematic diagram of the MNU-induced gastric cancer (GC) mouse model timeline, from induction to euthanasia (above). IHC images of SRSF10 in gastric dysplasia tissue and normal tissue of mice (below). **M** IHC images of SRSF10 in gastric cancer tissues from immunotherapy responder and non-responder. **N** Distribution of SRSF10 expression levels in responder and non-responder groups. **O** Kaplan–Meier overall survival (OS) curves comparing patients with high versus low SRSF10 expression. **P** Kaplan–Meier disease-free survival (DFS) curves comparing patients with high versus low SRSF10 expression. **Q** Univariate and multivariate Cox regression analyses for OS. *P < 0.05; **P < 0.01; ***P < 0.001. Scale bars in (**L**, **M**) 100 μm.

To investigate the heterogeneity of tumor cells in human gastric cancer, single-cell RNA sequencing (scRNA-seq) was performed on eighteen samples. Following the filtration of low-quality cells and the execution of dimensionality reduction and clustering, the cells were initially classified into 11 cell subpopulations based on marker genes (Fig. 1A and Supplementary Fig. 1A). Thereafter, we further subdivided epithelial cell subpopulations into 6 distinct subpopulations based on the expression of specific maker genes (Fig. 1B, C). Tumor-derived gastric epithelial cells exhibit significant differences from normal cells at the single-cell level. Density distributions reveal that normal tissue primarily consists of mature, differentiated surface epithelial cells and chief cells, whereas tumor tissue is enriched with metaplastic and dysplastic/tumor cell populations (Fig. 1D and Supplementary Fig. 1B, C).

Subsequently, to delineate regulatory networks governing differential responses to ICIs in GC, we interrogated transcriptome profiles between GC with different responses to ICIs. Leveraging the CIBERSORT algorithm [26], we uncovered distinct disparities in immune infiltrate composition, particularly diminished CD8⁺ T cell infiltration and pronounced M2-polarized macrophage accumulation in non-responders (Supplementary Fig. 1D). Subsequent gene set enrichment analysis (GSEA) using MSigDB Hallmark, Gene Ontology (GO), and KEGG databases revealed enrichment of immune response pathways, particularly involving mTORC1 signaling, spliceosome assembly, and RNA processing (Supplementary Fig. 1E, F). Strikingly, spliceosome components (SRSF/THOC families) were differentially upregulated in non-responders (Fig. 1E), implicating dysregulated RNA processing in T-cell exclusion and TAM polarization.

We next intersected the upregulated genes from scRNA-seq (Tumor vs. Normal) and bulk RNA-seq (Non-responder vs. Responder) with spliceosome-related genes, which yielded three consistently candidates: SRSF10, SRSF6 and THOC2 (Fig. 1F). Among these, SRSF10 demonstrated significantly higher expression in tumor tissues during scRNA-seq (Fig. 1G, H). Consistent with this, analysis of TCGA-STAD data confirmed elevated SRSF10 expression in tumor tissues (Fig. 1I). Notably, we identified significant dysregulation of spliceosome components, with SRSF10 demonstrating marked suppression in highly responsive tumors, suggesting its pivotal role in dictating ICIs response for GC patients (Fig. 1J, M, N). Subsequently, we employed immunohistochemistry (IHC) to observe the dynamic changes in SRSF10 expression across normal gastric tissue, intestinal metaplasia (IM), and dysplastic gastric tissue (Fig. 1K and Supplementary Fig. 2A). The results further confirmed that SRSF10 expression exhibits a stepwise increase throughout the entire gastric tumorigenesis process. qPCR results further confirmed elevated SRSF10 expression levels in gastric cancer tissues (Supplementary Fig. 2B). Assessing SRSF10 dynamics during gastric tumorigenesis, we employed N-methyl-N-nitrosourea (MNU)-induced chemical carcinogenesis, as previously reported [27–31]. We observed substantial SRSF10 upregulation during malignant transformation of gastric epithelium relative to adjacent non-neoplastic tissues (Fig. 1L).

Critically, elevated SRSF10 expression was associated with a poorer prognosis (Supplementary Fig. 2C), as indicated by reduced overall survival (OS; Fig. 1O) and disease-free survival (DFS; Fig. 1P). According to the univariate/multivariate Cox regression analyses, the BMI, TNM stage, and SRSF10 levels were significantly associated with both OS and DFS (Fig. 1Q and Supplementary Fig. 2D), implying, SRSF10 emerged as independent prognostic factors. These results reaffirm the stepwise increase of SRSF10 expression throughout gastric tumorigenesis. Collectively, these findings suggest SRSF10 may function as a modulator central to both the establishment of ICIs-refractory and gastric tumorigenesis, positioning it as a compelling therapeutic target.

### SRSF10 inhibition impairs growth of GC

**Fig. 2:**
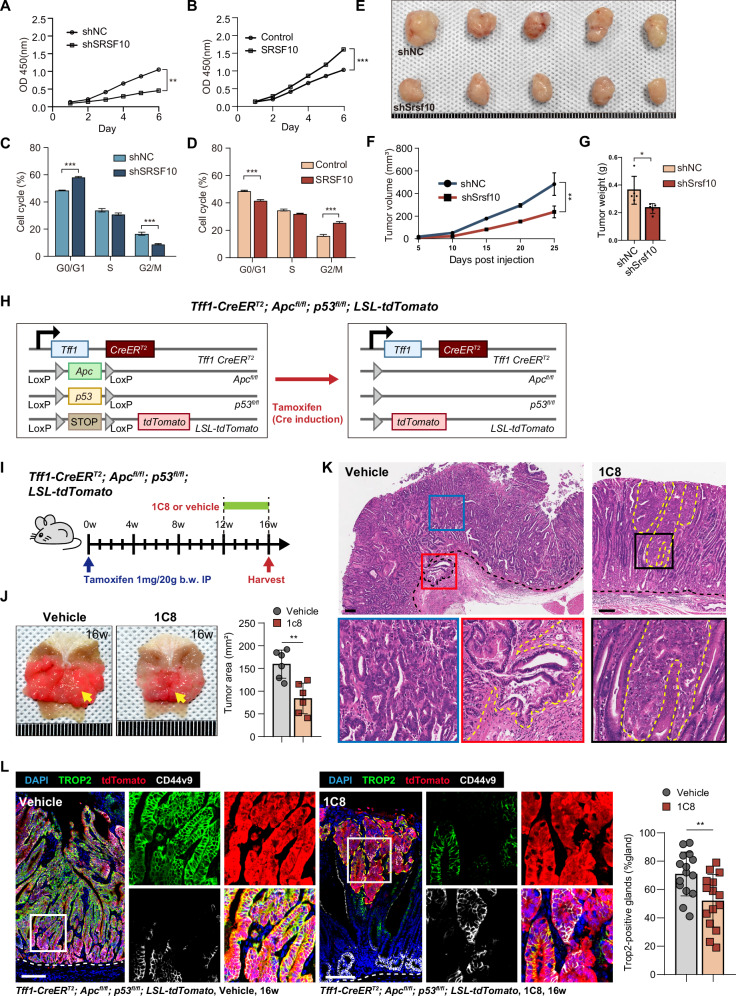
SRSF10 inhibition impairs growth of GC. **A** Cell proliferation curves of AGS-shNC and AGS-shSRSF10 cells assessed by CCK-8 assay. **B** Cell proliferation curves of AGS cells overexpressing SRSF10 and the control cells were assessed via a CCK-8 assay. **C** Cell cycle analysis of AGS-shNC and AGS-shSRSF10 cells by flow cytometry. **D** Flow cytometry was conducted to assess the cell cycle of AGS cells overexpressing SRSF10 and control cells. **E** Representative images of subcutaneous tumors from C57BL/6 wild-type mice injected with gastric cancer cells transduced with shNC or shSrsf10. **F** Growth curves of subcutaneous tumors derived from shNC and shSrsf10 cells in C57BL/6 wild-type mice. **G** Tumor weight comparison between shNC and shSrsf10 groups at the end of the experiment. **H** The experimental strategy for genetic recombination in the *Tff1-CreER*^*T*^^*2*^*; Apc*^*fl/fl*^*; p53*^*fl/fl*^*; LSL-tdTomato* mice model. **I** Tamoxifen and 1C8 treatment schematic. **J** Macroscopic images of gastric tumors in 1C8 and control-treated mice are shown in the figure (left). The total area occupied by gastric tumors in each mouse is shown in the graph (right). **K** Representative images and haematoxylin and eosin (H&E) staining of gastric cancer nodules in *Tff1-CreER*^*T2*^*; Apc*^*fl/fl*^*; p53*^*fl/fl*^*; LSL-tdTomato* mice, 16 weeks after infection with tamoxifen and 1C8, or the control treatment. (**L**) Representative images and quantitative analysis of TROP2^+^ cells in the gastric tissue of mice that were treated with either 1C8 or the control treatment(left). The proportion of TROP2^+^ glands in all gastric glands in the stomach tissues of mice receiving 1C8 or control treatment(right). *P < 0.05; **P < 0.01; ***P < 0.001. Scale bars in (**K**, **L**) 100 μm.

To further investigate the cell-autonomous effects of SRSF10 on GC progression, we established SRSF10 knockdown and overexpression cell lines in GC cells (Supplementary Fig. 3A, B). Utilizing CCK-8 proliferation assays and cell cycle analysis, we demonstrated that SRSF10 significantly enhances in vitro tumor cell proliferation, whereas its depletion exerts an inhibitory effect (Fig. 2A–D and Supplementary Fig. 3C, D). Subsequently, we established Srsf10 knockdown cell lines in YTN3 mice GC cells. In vivo xenograft models of C57BL/6 wild-type mice, tumors with Srsf10 knockdown exhibited markedly slower growth rates compared to control grafts (Fig. 2E–G).

Previously, Shkreta et al. identified the 4-pyridinone-benzisothiazole carboxamide compound 1C8 as a selective inhibitor of SRSF10 [24, 32], which mediates antiviral responses. Concurrently, Cai et al. demonstrated that targeting SRSF10 with 1C8 enhances MYB RNA stability, inhibits lactate production, and consequently augments the response to immunotherapy in hepatocellular carcinoma [25]. Building on this foundation, to investigate the role of SRSF10 inhibition in gastric cancerous events, we crossed *Tff1-CreER*^*T*^^*2*^ alleles with *Apc*^*fl/fl*^, *p53*^*fl/fl*^, and *LSL-tdTomato* mice to generate gastric adenocarcinoma models [33], thereby assessing the functional impact of SRSF10 inhibition via 1C8 on tumorigenesis (Fig. 2H, I). Notably, gastric tumor areas in 1C8-treated mice were significantly smaller than those in controls (Fig. 2J). Intriguingly, submucosal tumor cell infiltration was observed in a subset of control mice after 16 weeks of induction, whereas this phenomenon was entirely absent in 1C8-treated mice (Fig. 2K). To delineate the role of SRSF10 suppression in gastric carcinogenesis, we employed Trop2 as a marker for dysplastic cells. Immunofluorescence analysis revealed robust Trop2 expression in the majority of glands in control mice, contrasting with a reduction in Trop2-positive dysplastic cells in 1C8-treated mice (Fig. 2L). Thus, SRSF10 propels gastric carcinogenesis by fueling proliferation and dysplastic advancement.

### Targeting SRSF10 reprograms the immunosuppressive niche via CCL2

**Fig. 3:**
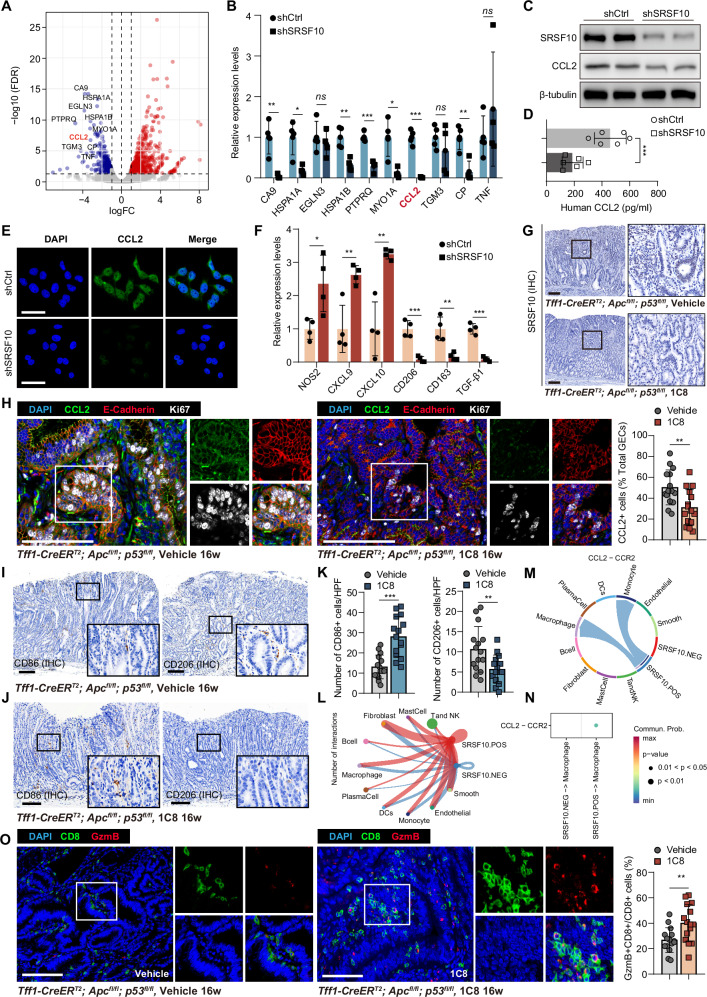
Targeting SRSF10 reprograms the immunosuppressive niche via CCL2. **A** A volcano plot displaying the results of gene expression differential analysis is produced by combining RNA-seq sequencing data from the shCtrl and shSRSF10 AGS cell lines. **B** The expression levels of mRNA for the top 10 differentially expressed genes were validated via real-time fluorescent quantitative reverse transcription polymerase chain reaction (RT-qPCR). **C** Western blotting demonstrates the impact of SRSF10 on the expression of the CCL2 protein. **D** ELISA demonstrates the impact of SRSF10 on the secretion of the CCL2 protein. **E** Cell Fluorescence showing the effect of SRSF10 on CCL2 protein expression. **F** Co-culturing SRSF10-knockdown or control gastric cancer cells with THP-1 cells revealed alterations in the expression of M1 and M2 macrophage markers in the THP-1 cells. **G** Immunohistochemistry (IHC) analysis of SRSF10 expression in gastric tissues from *Tff1-CreER*^*T*^^*2*^*; Apc*^*fl/fl*^*; p53*^*fl/fl*^*; LSL-tdTomato* mice treated with vehicle or 1C8. **H** Immunofluorescence staining of DAPI, CCL2, Ki67, and E-cadherin in gastric tissues from vehicle- or 1C8-treated mice(left). The percentage of CCL2^+^ cells among total GECs is shown(right). **I**, **J** Immunohistochemistry of CD206 (M2 macrophage marker) and CD86 (M1 macrophage marker) in gastric tissues from vehicle- or 1C8-treated mice. **K** Quantitative analysis of CD206^+^ cells/mm² and CD86^+^ cells/mm² in gastric cancer tissues. **L** The cell-cell interaction network reveals interactions between SRSF10-positive epithelial cells (red) and SRSF10-negative cells (blue) with immune cells (macrophages, T/NK cells) and mesenchymal cells (fibroblasts, endothelial cells, smooth muscle cells). **M** Schematic diagram of the CCL2-CCR2 signaling pathway, illustrating interactions between SRSF10-positive epithelial cells and macrophages. **N** Quantitative Analysis of CCL2-CCR2 Interaction Probability. **O** Immunofluorescence staining of CD8 and Granzyme B (GzmB) in gastric tissues from vehicle- or 1C8-treated mice. Quantification of GzmB⁺CD8⁺/CD8⁺ cell percentages in gastric cancer tissues. *P < 0.05; **P < 0.01; ***P < 0.001. Scale bars in (**E**), (**G**–**J**), (**O**), 100 μm.

To delineate the mechanistic basis of SRSF10-driven GC progression, we performed RNA-seq on SRSF10 knockdown GC cells. Differential gene expression analysis revealed that CCL2 (also known as MCP-1) was among the most significantly downregulated genes (Top 10) in the SRSF10 knockdown group (Fig. 3A). RT-qPCR further validated that CCL2 mRNA expression was significantly reduced upon SRSF10 depletion (Fig. 3B). Given CCL2’s established role in recruiting and polarizing M2-TAMs within the TME [34–36], we corroborated these findings through western blotting, ELISA, and immunofluorescence, collectively confirming diminished intracellular and secreted CCL2 protein (Fig. 3C–E). To ascertain whether SRSF10 influences the polarization of macrophages through CCL2, THP-1 monocytes were co-cultured with either shSRSF10 or control GC cells. Strikingly, macrophages exposed to shSRSF10 cells exhibited marked suppression of M2 markers (CD206, CD163, TGF-β1) and concomitant upregulation of M1 markers (NOS2, CXCL9, CXCL10) (Fig. 3F), indicating SRSF10 loss reprograms macrophages toward pro-inflammatory phenotypes.

Importantly, we crossed *Tff1-CreER*^*T*^^*2*^ alleles with *Apc*^*fl/fl*^ and *p53*^*fl/fl*^ mice to establish a gastric adenocarcinoma model to assess the effects of SRSF10 in TME using a selective inhibitor 1C8. We confirmed that 1C8 efficiently inhibits SRSF10 expression in *Tff1-CreER*^*T*^^*2*^*; Apc*^*fl/fl*^*; p53*^*fl/fl*^ orthotopic GC models (Fig. 3G). The immunofluorescence results revealed substantially diminished CCL2 expression in the 1C8-treated group than in the control group (Fig. 3H). Consistently, IHC revealed expanded CD86⁺ M1-macrophages and contracted CD206⁺ M2-macrophages populations (Fig. 3I–K). To further investigate the communication patterns between SRSF10-positive epithelial cells and the tumor microenvironment, Cell Chat analysis was performed. The results of the study indicated a substantial increase in the interactions between SRSF10-positive cells and various immune or stromal cell types, including fibroblasts, endothelial cells, and macrophages (Fig. 3L). A notable finding was the observation of substantial ligand-receptor interactions between SRSF10-positive epithelial cells and macrophages via the CCL2–CCR2 signaling pathway (Fig. 3M). Quantitative analysis further confirmed that CCL2–CCR2 interactions were significantly enhanced in SRSF10-positive epithelial cells compared to SRSF10-negative cells (Fig. 3N). Further immunofluorescence results revealed significantly enhanced recruitment of GzmB⁺/CD8⁺ T cells in the 1C8-treated group compared to the control group (Fig. 3O). Collectively, these data establish that SRSF10 inhibition reprograms macrophages in the immunosuppressive TME by ablating CCL2.

### SRSF10 blockade attenuates pre-neoplastic metaplastic cell proliferation and TAMs reprogramming

**Fig. 4:**
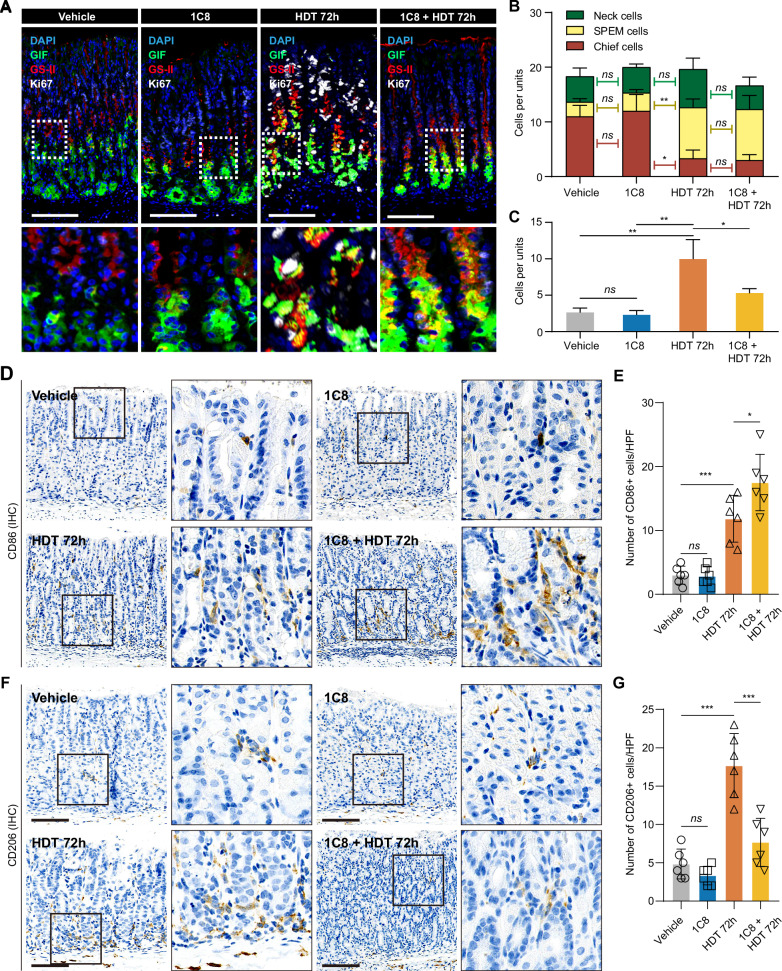
SRSF10 blockade attenuates pre-neoplastic metaplastic cell proliferation and TAMs reprogramming. **A** Representative immunofluorescence staining of mouse gastric tissues for DAPI, GIF, GS-II, and Ki67 across four treatment groups: vehicle, 1C8, HDT 72 h, and 1C8 + HDT 72 h. **B** Quantification of chief cells, neck cells, and SPEM cells per gastric unit in mice treated with vehicle, 1C8, HDT 72 h, or combination of 1C8 + HDT 72 h. **C** Quantification of Ki67⁺GIF⁺ proliferating chief cells and Ki67⁺GS-II⁺ proliferating SPEM cells per gastric unit under different treatment conditions. **D** Representative IHC staining images of CD86 in Vehicle, 1C8, HDT 72 h, and 1C8 + HDT 72 h groups. **E** Quantification of CD86^+^ cells/HPF in different groups. **F** Representative IHC staining images of CD206 in Vehicle, 1C8, HDT 72 h, and 1C8 + HDT 72 h groups. **G** Quantification of CD206^+^ cells/HPF in different groups. *P < 0.05; **P < 0.01; ***P < 0.001. Scale bars in (**A**),100 μm.

To further investigate SRSF10’s role in pre-neoplastic metaplastic process, we employed a high-dose tamoxifen (HDT) injury model, which has been extensively utilized by us and others [30, 37–39]. HDT induces the loss of acid-secreting parietal cells in the gastric corpus and induces the formation of proliferative metaplastic cells (Supplementary Fig. 4A), has been called pseudopyloric metaplasia or spasmolytic polypeptide expressing metaplasia (SPEM) [37, 38, 40]. Notably, 1C8 monotherapy in the naive stomach exerted negligible effects on isthmus mitotic activity (Fig. 4A–C). Intriguingly, while 1C8 failed to disrupt SPEM initiation, it selectively abrogated the proliferative capacity of HDT-induced SPEM-like cells (Fig. 4A–C). Immunohistochemical profiling demonstrated that 1C8 significantly reprogrammed the TAM landscape: contracting CD206⁺ M2-like populations while expanding CD86⁺ M1-like macrophages (Fig. 4D–G). This polarization shift implies SRSF10 inhibition attenuates pre-neoplastic metaplastic immunosuppressive niche. To evaluate SRSF10 expression in the HDT-induced SPEM model, we performed Western blotting and immunohistochemistry. Western blotting showed significantly higher SRSF10 protein levels in the HDT group than in controls (Supplementary Fig. 4B). Immunohistochemical quantification revealed an increased number of SRSF10⁺ epithelial cells per HPF in the HDT group (Supplementary Fig. 4C, D).

### SRSF10 regulates BCAT2 exon skipping to activates mTORC1 signaling

**Fig. 5:**
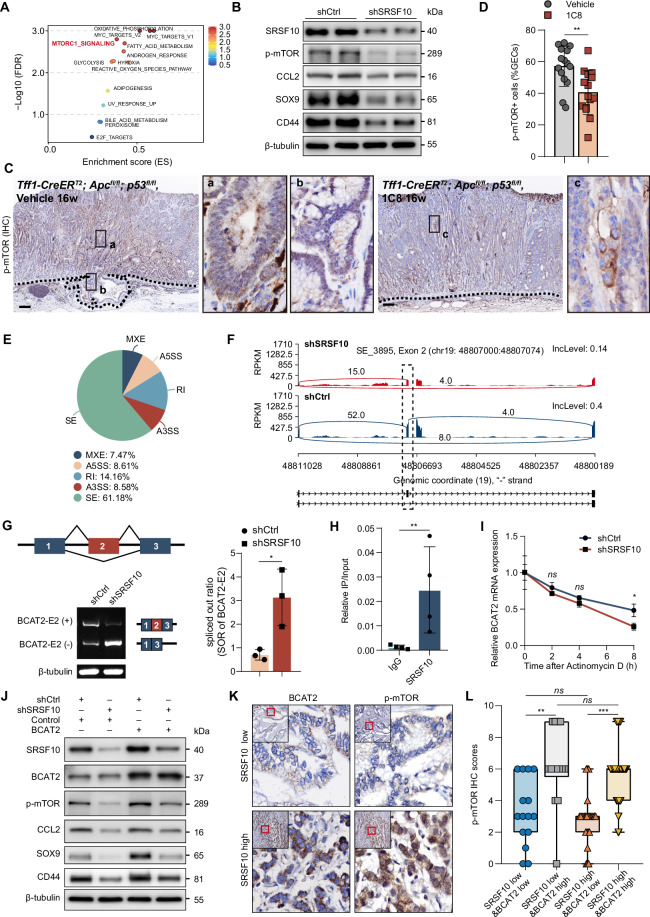
SRSF10 regulates BCAT2 exon skipping to activates mTORC1 signaling. **A** Gene Set Enrichment Analysis (GSEA) of hallmark pathways in AGS cells upon SRSF10 knockdown. **B** Western blot analysis showing the expression levels of SRSF10, CD44, SOX9, CCL2, BCAT2, and p-mTOR in AGS cells with or without SRSF10 knockdown. **C** Immunohistochemistry (IHC) analysis of p-mTOR expression in gastric tissues from *Tff1-CreER*^*T*^^*2*^*; Apc*^*fl/fl*^*; p53*^*fl/fl*^*; LSL-tdTomato* mice treated with vehicle or 1C8. The dotted line denotes the boundary of the mucosal muscularis layer, whilst the black boxes a–c represent high-power fields of view. **D** Proportion of p-mTOR^+^ cells in GECs under Vehicle or 1C8 treatment. **E** Percentages of AS events (SE, MXE, A5SS, A3SS, RI) detected by rMATS analysis in SRSF10-knockdown versus control cells. **F** Sashimi plot showing BCAT2 exon 2 (chr19: 48807000–48807074) inclusion rates, decreased in shSRSF10 compared to shCtrl. Arcs indicate junction reads. **G** RT-PCR validation of BCAT2-E2(+) and BCAT2-E2(−) isoforms in control and SRSF10-knockdown cells(left). Spliced-out ratio (SOR) of BCAT2-E2 in shCtrl versus shSRSF10 cells(right). **H** RNA immunoprecipitation (RIP). **I** BCAT2 mRNA decay curves in shCtrl and shSRSF10 AGS cells following Actinomycin D treatment (0–8 h). **J** Western blot analysis of SRSF10, BCAT2, p-mTOR, CCL2, SOX9 and CD44 in gastric cancer cells under different conditions (shCtrl, shSRSF10, Control, BCAT2). **K** Immunohistochemistry (IHC) staining of BCAT2 and p-mTOR in gastric cancer tissues with low and high SRSF10 expression. **L** Box plots showing the p-mTOR HC scores in different groups based on SRSF10 and BCAT2 expression levels. *P < 0.05; **P < 0.01; ***P < 0.001. Scale bars in (**C**, **K**) 100 μm.

To elucidate SRSF10’s mechanistic link to CCL2, we bioinformatically scanned the CCL2 pre-mRNA (ENST000002258314) for potential binding sites using the RBP suite algorithm [41]. However, no clear evidence was found to support a direct interaction between SRSF10 and CCL2 mRNA (Supplementary Fig. 5A). Intriguingly, transcriptome-wide GSEA revealed pronounced suppression of mTORC1 signaling upon SRSF10 knockdown (Fig. 5A and Supplementary Fig. 5B). Corroborating this, western blot demonstrated concerted attenuation of p-mTOR, CCL2, and stemness factors SOX9/CD44 (Fig. 5B). Immunohistochemistry revealed widespread p-mTOR expression in gastric epithelial cells of control mice, with particularly strong staining observed in dysplastic tissues. Treatment with the SRSF10 inhibitor 1C8 markedly reduced both the staining intensity and proportion of p-mTOR-positive epithelial cells (Fig. 5C). Quantitative image analysis confirmed that 1C8 treatment significantly decreased the proportion of p-mTOR-positive epithelial cells (Fig. 5D). Using the *Tff1-CreER*^*T*^^*2*^*; Apc*^*fl/fl*^*; p53*^*fl/fl*^ mice models, we confirmed reduced p-mTOR⁺ gastric epithelial cells (GECs) with 1C8 treatment compared to control group (Fig. 5C, D), validating the mTORC1 inhibition by SRSF10 ablation.

To identify the AS events regulating mTOR signaling, we intersected genes that underwent AS (junction count-only method) with differential expression genes (DEGs; *P* < 0.05, LogFC < −1). We found 13 AS events (Fig. 5E and Supplementary Fig. 5C). Among these AS events, BCAT2 have been reported to activate the mTOR signaling [42, 43]. Encouragingly, SRSF10 knockdown led to an increased exclusion of exon 2, generating more BCAT2 transcripts with exon 2 exclusion [BCAT2-E2(-)] and fewer BCAT2 transcripts with exon 2 inclusion [BCAT2-E2(+)] compared with the control group (Fig. 5F, G). Notably, RIP established direct SRSF10 binding to BCAT2 pre-mRNA (Fig. 5H), while actinomycin D assays proved SRSF10 stabilizes BCAT2 transcripts (Fig. 5I). Critically, BCAT2 overexpression rescued p-mTOR, CCL2, SOX9, and CD44 expression (Fig. 5J), affirming functional causality. IHC of human GC tissues revealed SRSF10 levels correlated positively with BCAT2 but relatively weakly correlated with p-mTOR (Supplementary Fig. 5D). To this end, we further found minimal p-mTOR in SRSF10-low&BCAT2-low specimens versus maximal activation in SRSF10-high&BCAT2-high cohorts (Fig. 5K, L), indicating that the activation of p-mTOR by SRSF10 is dependent on BCAT2.

### Pharmacological antagonism of SRSF10 potentiate anti-PD-1 therapy efficacy

**Fig. 6:**
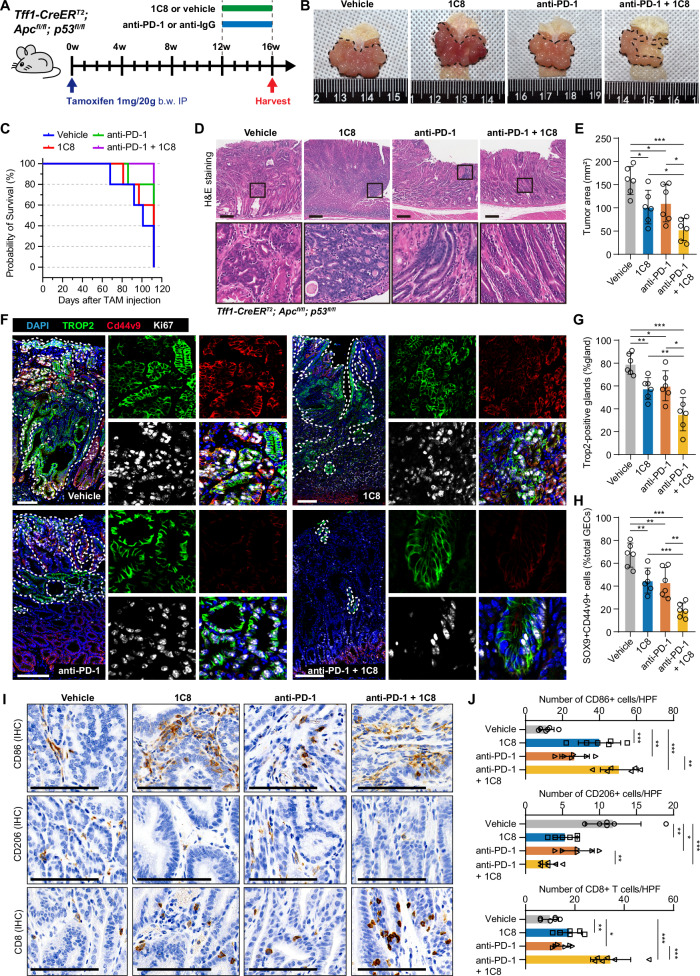
Pharmacological antagonism of SRSF10 potentiate anti-PD-1 therapy efficacy. **A** Experimental timeline and drug administration diagram for the *Tff1-CreER*^*T*^^*2*^*; Apc*^*fl/fl*^*; p53*^*fl/fl*^*; LSL-tdTomato* mice model. **B** Macroscopic tumor morphology comparison of gastric tissue from mice in different treatment groups. **C** Kaplan–Meier survival curves of mice in each treatment group. **D** H&E-stained sections of gastric tissue were examined from each group of mice. **E** Quantitative analysis of tumor area in different treatment groups. **F** Immunofluorescence staining images of gastric tissue from mice in different treatment groups (vehicle, 1C8, anti-PD-1 and anti-PD-1 + 1C8). TROP2 (green), CD44v9 (red), Ki67 (white) and DAPI (blue). **G** Quantitative analysis of the proportion of TROP2-positive glands. **H** The proportion of SOX9^+^ CD44v9^+^double-positive cells in total GECs. **I** Immunohistochemistry (IHC) analysis of CD86, CD206 and CD8 expression in gastric tissues from mice in different treatment groups (vehicle, 1C8, anti-PD-1 and anti-PD-1 + 1C8). **J** Quantification of CD86⁺ M1-like macrophages per high-power field (HPF)(above). Quantification of CD206⁺ M2-like macrophages per HPF (in between). Quantification of CD8^+^ T cells per HPF (below). *P < 0.05; **P < 0.01; ***P < 0.001. Scale bars in (**D**, **F**, **I**) 100 μm.

We confirmed that SRSF10 inhibition could increase the infiltration of CD8^+^ T cells and reprogram TAMs and convert them into M1-like TAMs in the TME. To investigate whether targeting SRSF10 enhances the therapeutic effect of anti-PD-1, we administered PD-1 monoclonal antibodies, SRSF10-targeting agent 1C8, IgG in *Tff1-CreER*^*T*^^*2*^*; Apc*^*fl/fl*^*; p53*^*fl/fl*^ mice by administering tamoxifen (Fig. 6A). The results revealed that the combination therapy of 1C8 and anti-PD-1 significantly reduced gastric tumor burden compared to monotherapy or control treatment alone (Fig. 6B–E). Notably, survival analysis showed that GC-burdened mice receiving the combined treatment exhibited a significant survival advantage over all other treatment groups (Fig. 6C).

We further conducted Immunofluorescence evaluation of TROP2 (marker of intramucosal dysplasia) and CD44v9/SOX9 (marker for metaplastic lineage) to elucidate alterations in metaplastic or dysplastic cells following combination therapy. Notably, the combined regimen of 1C8 and anti-PD-1 antibody demonstrated significant efficacy in suppressing the emergence of TROP2^+^ dysplastic cells (Fig. 6F, G), concomitant with a marked reduction in SOX9^+^/CD44v9^+^ metaplastic cell populations (Fig. 6H and Supplementary Fig. 6A).

The effect of the combination therapy of 1C8 and anti-PD-1 on macrophage phenotype was evaluated by IHC. Our data showed that more cells expressed CD86^+^ M1-type TAMs, but less cells expressed CD206^+^ M2-type TAMs in gastric tissue in combined regimen groups (Fig. 6I–J). As expected, combination treatment synergistically promoted intra tumoral CD8^+^ T cells expansion in *Tff1-CreER*^*T*^^*2*^*; Apc*^*fl/fl*^*; p53*^*fl/fl*^ mice. Collectively, our data revealed that SRSF10 is a key target of the immunotherapy response in GC, and provided proof of concept the blockade of SRSF10 is a potentially effective approach for improving the efficacy of immunotherapy in GC.

## Discussion

Advanced GC remains a lethal disease with a poor ICIs response and prognosis, necessitating urgent elucidation of the mechanisms underlying its immune escape, and developing effective therapeutic strategies [5, 44, 45]. Recent studies have revealed the pivotal function of AS in tumor progression and immune regulation [21], yet the role of AS and immunotherapy response in GC remains unclear. Our study establishes splicing factor SRSF10 as a master regulator orchestrating both tumor-intrinsic growth and immunosuppressive niche formation. We revealed that SRSF10 regulates exon skipping of the BCAT2 gene, thereby influencing mTOR activation and CCL2-mediated macrophage polarization, and ultimately promoting immune escape and tumor progression. Pharmacological antagonism of SRSF10 synergized with anti-PD-1 by reshaping macrophage plasticity, proposing a druggable strategy to overcome GC immunotherapy resistance.

Transcriptomic profiling of ICIs responders versus non-responders revealed SRSF10 as one of the most differentially expressed spliceosome component, exhibiting pronounced elevation in therapy-resistant GC. Critically, its progressive upregulation during the metaplasia-dysplasia-carcinoma sequence, suggesting SRSF10 acts a duality gene by potentiating both malignant transformation and immune escape. This aligns with emerging evidence that spliceosome generate neoantigens while paradoxically fostering T-cell exhaustion [46, 47]. Notably, SRSF10 expression inversely correlated with GC patient survival outcomes, establishing it as an independent prognostic factor—a finding consistent with its oncogenic roles in hepatocellular carcinoma and cervical cancer [24, 25].

A vital revelation of our study is the SRSF10-CCL2 axis as a driver of TAM polarization. SRSF10 knockdown drastically reduced CCL2 transcription and secretion, shifting macrophages from immunosuppressive M2 (CD206⁺/CD163⁺/TGF-β⁺) to pro-inflammatory M1 phenotypes (CD86⁺/CXCL9⁺/NOS2⁺). This reprogramming enhanced intratumoral CD8⁺ T-cell infiltration and granzyme B production, effectively converting immune-deserted tumors into immunoreactive niches. CCL2 (MCP-1) is a well-established chemokine recruiting CCR2⁺ monocytes and polarizing them toward M2 states [48]. Our data extend this paradigm by implicating SRSF10 as an upstream transcriptional regulator of CCL2, thereby positioning splicing machinery as a key modulator of chemokine-driven immune evasion.

Intriguingly, we revealed that SRSF10’s regulation of CCL2 is indirect, mediated through mTORC1 activation via BCAT2 exon 2 inclusion. BCAT2, a metabolic enzyme in branched-chain amino acid (BCAA) catabolism, has been reported to activate mTORC1 in colorectal cancer progression [49]. We demonstrated that SRSF10 binds BCAT2 pre-mRNA, enforcing exon 2 inclusion [BCAT2-E2(+)] and enhancing transcript stability.

Harnessing innate antitumor immunity has emerged as a pivotal therapeutic component of combinatorial immunotherapy regimens [50]. In this study, we showed that pharmacologically targeting SRSF10, using a small-molecule inhibitor 1C8, significantly reduced tumor burden and improved survival in a spontaneous GC mouse model. The combination therapy not only suppressed tumor cell proliferation but also further reduced expression of SOX9 and CD44v9, suggesting that dual targeting of SRSF10 and immune checkpoints may be an effective strategy to overcome resistance and eradicate stem-like tumor cells.

Emerging evidence indicates that SRSF10 in tumor cells is functionally connected to T cells [25, 51, 52]. Xia et al. and Zhou et al. reported that higher SRSF10 expression correlates with increased intratumoral CD8⁺ T-cell infiltration [51, 52]. Cai et al. further demonstrated that SRSF10 binds to MYB mRNA and stabilizes its expression, thereby activating glycolysis and promoting lactate accumulation, which drives macrophage polarization and suppresses CD8⁺ T-cell function in hepatocellular carcinoma [25]. In gastric cancer, our data show that SRSF10 inhibition reduces CCL2 secretion by regulating BCAT2 exon 2 alternative RNA splicing, thereby enhancing macrophage reprogramming and promoting intratumoral CD8⁺ T-cell infiltration. Current studies suggest that CCL2 recruits CCR2⁺ monocytes and drives their gradual conversion into M2-like TAMs [36, 53, 54], thereby weakening antitumor immunity. We acknowledge that our study provides initial insights into the role of SRSF10 in gastric cancer, but there are still limitations. Although we identified CCL2 secreted through SRSF10-regulated BCAT2 alternative RNA splicing, it remains unclear whether SRSF10 directly affects intratumoral CD8^+^ T cell infiltration independently of CCL2, which warrants deeper mechanistic and functional investigation.

SRSF10, a core member of the SR family of splicing factors, contains an RNA-recognition motif (RRM) that confers RNA-binding specificity and an arginine/serine-rich (RS) domain [55–57]. The RRM binds exonic and intronic splicing enhancers (ESEs/ISEs), with RNP1/RNP2 motifs at its N-terminus being essential for RNA interaction [56–59]. The RS domain mediates protein–protein interactions to assemble and regulate the spliceosome, and C-terminal SR/RS repeats facilitate cooperation with other splicing factors [55, 60]. Dysregulation or mutation of SRSF10 in cancer can promote tumorigenesis by altering pre-mRNA splicing, cellular metabolism, mRNA decay, and translation [58, 61, 62]. Functionally, SRSF10 plays critical roles in tumors. In cervical cancer, studies report that SRSF10 mediates IL1RAP alternative splicing, enhances the IL-1β/NF-κB signaling pathway, and upregulates CD47, thereby inhibiting macrophage phagocytosis and promoting tumorigenesis [24]. In hepatocellular carcinoma, SRSF10 promotes M2 macrophage polarization and accelerates tumor progression [25]. Our study reveals that in gastric cancer, SRSF10 drives BCAT2 exon skipping to activate the mTORC1 signaling pathway, thereby increasing CCL2 secretion to recruit macrophages and ultimately weakening antitumor immunity.

In conclusion, our study provides insight into the prominent role of SRSF10 in reprograming the tumor immunosuppressive niche, driven by BCAT2-dependent mTOR activation and CCL2-mediated TAMs polarization. Pharmacological inhibition of SRSF10 reshapes TAMs and synergizes with PD-1 blockade, providing a compelling rationale for clinical translation. As aberrant splicing emerges as a hallmark of cancer immune-resistance, targeting spliceosome nodes like SRSF10 offers a precision approach to reignite antitumor immunity.

## Supplementary information

Supplemental methods, figures and tables

Full length western blots

## Data Availability

The data used in this paper have been deposited in the OMIX repository at China National Center for Bioinformation/Beijing Institute of Genomics, Chinese Academy of Sciences (no. OMIX013154 and no. OMIX013158). The data supporting the findings of this study are available from the corresponding author on request.

## References

[1] Ribas A, Wolchok JD. Cancer immunotherapy using checkpoint blockade. Science. 2018;359:1350–5. 29567705 10.1126/science.aar4060 PMC7391259

[2] Chen DS, Mellman I. Elements of cancer immunity and the cancer-immune set point. Nature. 2017;541:321–30. 28102259 10.1038/nature21349

[3] He X, Xu C. Immune checkpoint signaling and cancer immunotherapy. Cell Res. 2020;30:660–9. 32467592 10.1038/s41422-020-0343-4 PMC7395714

[4] Cristescu R, Mogg R, Ayers M, Albright A, Murphy E, Yearley J, et al. Pan-tumor genomic biomarkers for PD-1 checkpoint blockade–based immunotherapy. Science. 2018;362:eaar3593. 30309915 10.1126/science.aar3593 PMC6718162

[5] Cristescu R, Lee J, Nebozhyn M, Kim KM, Ting JC, Wong SS, et al. Molecular analysis of gastric cancer identifies subtypes associated with distinct clinical outcomes. Nat Med. 2015;21:449–56. 25894828 10.1038/nm.3850

[6] Shitara K, Van Cutsem E, Bang YJ, Fuchs C, Wyrwicz L, Lee KW, et al. Efficacy and safety of pembrolizumab or pembrolizumab plus chemotherapy vs chemotherapy alone for patients with first-line, advanced gastric cancer: the KEYNOTE-062 phase 3 randomized clinical trial. JAMA Oncol. 2020;6:1571–80. 32880601 10.1001/jamaoncol.2020.3370 PMC7489405

[7] Janjigian YY, Shitara K, Moehler M, Garrido M, Salman P, Shen L, et al. First-line nivolumab plus chemotherapy versus chemotherapy alone for advanced gastric, gastro-oesophageal junction, and oesophageal adenocarcinoma (CheckMate 649): a randomised, open-label, phase 3 trial. Lancet. 2021;398:27–40. 34102137 10.1016/S0140-6736(21)00797-2 PMC8436782

[8] Smyth EC, Nilsson M, Grabsch HI, van Grieken NCT, Lordick F. Gastric cancer. Lancet. 2020;396:635–48. 32861308 10.1016/S0140-6736(20)31288-5

[9] Wang D, Zhang J, Wang J, Cai Z, Jin S, Chen G. Identification of collagen subtypes of gastric cancer for distinguishing patient prognosis and therapeutic response. Cancer Innov. 2024;3:e125. 38948250 10.1002/cai2.125 PMC11212290

[10] Noy R, Pollard JW. Tumor-associated macrophages: from mechanisms to therapy. Immunity. 2014;41:49–61. 25035953 10.1016/j.immuni.2014.06.010 PMC4137410

[11] Mantovani A, Allavena P, Marchesi F, Garlanda C. Macrophages as tools and targets in cancer therapy. Nat Rev Drug Discov. 2022;21:799–820. 35974096 10.1038/s41573-022-00520-5 PMC9380983

[12] Mantovani A, Marchesi F, Malesci A, Laghi L, Allavena P. Tumour-associated macrophages as treatment targets in oncology. Nat Rev Clin Oncol. 2017;14:399–416. 28117416 10.1038/nrclinonc.2016.217 PMC5480600

[13] Murray PJ, Allen JE, Biswas SK, Fisher EA, Gilroy DW, Goerdt S, et al. Macrophage activation and polarization: nomenclature and experimental guidelines. Immunity. 2014;41:14–20. 25035950 10.1016/j.immuni.2014.06.008 PMC4123412

[14] DeNardo DG, Ruffell B. Macrophages as regulators of tumour immunity and immunotherapy. Nat Rev Immunol. 2019;19:369–82. 30718830 10.1038/s41577-019-0127-6 PMC7339861

[15] Vervoort SJ, Devlin JR, Kwiatkowski N, Teng M, Gray NS, Johnstone RW. Targeting transcription cycles in cancer. Nat Rev Cancer. 2022;22:5–24. 34675395 10.1038/s41568-021-00411-8

[16] Sveen A, Kilpinen S, Ruusulehto A, Lothe RA, Skotheim RI. Aberrant RNA splicing in cancer; expression changes and driver mutations of splicing factor genes. Oncogene. 2016;35:2413–27. 26300000 10.1038/onc.2015.318

[17] Baralle FE, Giudice J. Alternative splicing as a regulator of development and tissue identity. Nat Rev Mol Cell Biol. 2017;18:437–51. 28488700 10.1038/nrm.2017.27 PMC6839889

[18] Wang Y, Bao Y, Zhang S, Wang Z. Splicing dysregulation in cancer: from mechanistic understanding to a new class of therapeutic targets. Sci China Life Sci. 2020;63:469–84. 32086672 10.1007/s11427-019-1605-0

[19] Lee SC, Abdel-Wahab O. Therapeutic targeting of splicing in cancer. Nat Med. 2016;22:976–86. 27603132 10.1038/nm.4165 PMC5644489

[20] Patowary A, Zhang P, Jops C, Vuong CK, Ge X, Hou K, et al. Developmental isoform diversity in the human neocortex informs neuropsychiatric risk mechanisms. Science. 2024;384:eadh7688. 38781356 10.1126/science.adh7688 PMC11960787

[21] Dvinge H, Kim E, Abdel-Wahab O, Bradley RK. RNA splicing factors as oncoproteins and tumour suppressors. Nat Rev Cancer. 2016;16:413–30. 27282250 10.1038/nrc.2016.51 PMC5094465

[22] Bei M, Xu J. SR proteins in cancer: function, regulation, and small inhibitor. Cell Mol Biol Lett. 2024;29:78. 38778254 10.1186/s11658-024-00594-6 PMC11110342

[23] Kumar K, Sinha SK, Maity U, Kirti PB, Kumar KRR. Insights into established and emerging roles of SR protein family in plants and animals. Wiley Interdiscip Rev RNA. 2023;14:e1763. 36131558 10.1002/wrna.1763

[24] Liu F, Dai M, Xu Q, Zhu X, Zhou Y, Jiang S, et al. SRSF10-mediated IL1RAP alternative splicing regulates cervical cancer oncogenesis via mIL1RAP-NF-κB-CD47 axis. Oncogene. 2018;37:2394–409. 29429992 10.1038/s41388-017-0119-6 PMC5931977

[25] Cai J, Song L, Zhang F, Wu S, Zhu G, Zhang P, et al. Targeting SRSF10 might inhibit M2 macrophage polarization and potentiate anti-PD-1 therapy in hepatocellular carcinoma. Cancer Commun. 2024;44:1231–60. 10.1002/cac2.12607 PMC11570766 39223929

[26] Newman AM, Liu CL, Green MR, Gentles AJ, Feng W, Xu Y, et al. Robust enumeration of cell subsets from tissue expression profiles. Nat Methods. 2015;12:453–7. 25822800 10.1038/nmeth.3337 PMC4739640

[27] Tatematsu M, Yamamoto M, Shimizu N, Yoshikawa A, Fukami H, Kaminishi M, et al. Induction of glandular stomach cancers in Helicobacter pylori-sensitive Mongolian gerbils treated with N-methyl-N-nitrosourea and N-methyl-N’-nitro-N-nitrosoguanidine in drinking water. Jpn J Cancer Res. 1998;89:97–104. 9548434 10.1111/j.1349-7006.1998.tb00535.x PMC5921771

[28] Tatematsu M, Ogawa K, Hoshiya T, Shichino Y, Kato T, Imaida K, et al. Induction of adenocarcinomas in the glandular stomach of BALB/c mice treated with N-Methyl-N-nitrosourea. Jpn J Cancer Res. 1992;83:915–8. 1429199 10.1111/j.1349-7006.1992.tb01999.x PMC5918972

[29] Sugiyama A, Maruta F, Ikeno T, Ishida K, Kawasaki S, Katsuyama T, et al. Helicobacter pylori infection enhances N-methyl-N-nitrosourea-induced stomach carcinogenesis in the Mongolian gerbil. Cancer Res. 1998;58:2067–9. 9605743

[30] Huang XB, Huang Q, Jiang MC, Zhong Q, Zheng HL, Wang JB, et al. KLHL21 suppresses gastric tumourigenesis via maintaining STAT3 signalling equilibrium in stomach homoeostasis. Gut. 2024;73:1785–98. 38969490 10.1136/gutjnl-2023-331111

[31] Miao ZF, Sun JX, Adkins-Threats M, Pang MJ, Zhao JH, Wang X, et al. DDIT4 licenses only healthy cells to proliferate during injury-induced metaplasia. Gastroenterology. 2021;160:260–71.e10. 32956680 10.1053/j.gastro.2020.09.016 PMC7857017

[32] Shkreta L, Blanchette M, Toutant J, Wilhelm E, Bell B, Story BA, et al. Modulation of the splicing regulatory function of SRSF10 by a novel compound that impairs HIV-1 replication. Nucleic Acids Res. 2017;45:4051–67. 27928057 10.1093/nar/gkw1223 PMC5397194

[33] Zhong Q, Wang HG, Yang JH, Tu RH, Li AY, Zeng GR, et al. Loss of ATOH1 in pit cell drives stemness and progression of gastric adenocarcinoma by activating AKT/mTOR signaling through GAS1. Adv Sci. 2023;10:e2301977. 10.1002/advs.202301977 PMC10646280 37824217

[34] Qian B-Z, Pollard JW. Macrophage diversity enhances tumor progression and metastasis. Cell. 2010;141:39–51. 20371344 10.1016/j.cell.2010.03.014 PMC4994190

[35] Li X, Yao W, Yuan Y, Chen P, Li B, Li J, et al. Targeting of tumour-infiltrating macrophages via CCL2/CCR2 signalling as a therapeutic strategy against hepatocellular carcinoma. Gut. 2017;66:157–67. 26452628 10.1136/gutjnl-2015-310514

[36] Pozzi S, Satchi-Fainaro R. The role of CCL2/CCR2 axis in cancer and inflammation: The next frontier in nanomedicine. Adv Drug Deliv Rev. 2024;209:115318. 38643840 10.1016/j.addr.2024.115318

[37] Hata M, Kinoshita H, Hayakawa Y, Konishi M, Tsuboi M, Oya Y, et al. GPR30-expressing gastric chief cells do not dedifferentiate but are eliminated via PDK-dependent cell competition during development of metaplasia. Gastroenterology. 2020;158:1650–66.e15. 32032583 10.1053/j.gastro.2020.01.046 PMC8796250

[38] Hayakawa Y, Fox JG, Wang TC. Isthmus stem cells are the origins of metaplasia in the gastric corpus. Cell Mol Gastroenterol Hepatol. 2017;4:89–94. 28560293 10.1016/j.jcmgh.2017.02.009 PMC5440357

[39] Shah SC, Gawron AJ, Li D. Surveillance of gastric intestinal metaplasia. Off J Am Coll Gastroenterol. 2020;115:641–4. 10.14309/ajg.0000000000000540 PMC7364865 32058339

[40] Shimizu T, Choi E, Petersen CP, Noto JM, Romero-Gallo J, Piazuelo MB, et al. Characterization of progressive metaplasia in the gastric corpus mucosa of Mongolian gerbils infected with Helicobacter pylori. J Pathol. 2016;239:399–410. 27125972 10.1002/path.4735 PMC4958595

[41] Pan X, Fang Y, Liu X, Guo X, Shen HB. RBPsuite 2.0: an updated RNA-protein binding site prediction suite with high coverage on species and proteins based on deep learning. BMC Biol. 2025;23:74. 40069726 10.1186/s12915-025-02182-2 PMC11899677

[42] Wang X, Xu J, Zeng H, Han Z. Enhancement of BCAT2-mediated valine catabolism stimulates β-casein synthesis via the AMPK-mTOR signaling axis in bovine mammary epithelial cells. J Agric Food Chem. 2022;70:9898–907. 35916279 10.1021/acs.jafc.2c03629

[43] Dhanani ZN, Mann G, Adegoke OAJ. Depletion of branched-chain aminotransferase 2 (BCAT2) enzyme impairs myoblast survival and myotube formation. Physiol Rep. 2019;7:e14299. 31833233 10.14814/phy2.14299 PMC6908738

[44] Binnewies M, Roberts EW, Kersten K, Chan V, Fearon DF, Merad M, et al. Understanding the tumor immune microenvironment (TIME) for effective therapy. Nat Med. 2018;24:541–50. 29686425 10.1038/s41591-018-0014-x PMC5998822

[45] Cassetta L, Pollard JW. Targeting macrophages: therapeutic approaches in cancer. Nat Rev Drug Discov. 2018;17:887–904. 30361552 10.1038/nrd.2018.169

[46] Anczukow O, Allain FH, Angarola BL, Black DL, Brooks AN, Cheng C, et al. Steering research on mRNA splicing in cancer towards clinical translation. Nat Rev Cancer. 2024;24:887–905. 39384951 10.1038/s41568-024-00750-2 PMC11698124

[47] Wang J, Yan L, Wang X, Jia R, Guo J. Surface PD-1 expression in T cells is suppressed by HNRNPK through an exonic splicing silencer on exon 3. Inflamm Res. 2024;73:1123–35. 38698180 10.1007/s00011-024-01887-4

[48] Weng J, Wang Z, Hu Z, Xu W, Sun JL, Wang F, et al. Repolarization of immunosuppressive macrophages by targeting SLAMF7-regulated CCL2 signaling sensitizes hepatocellular carcinoma to immunotherapy. Cancer Res. 2024;84:1817–33. 38484085 10.1158/0008-5472.CAN-23-3106

[49] Kang ZR, Jiang S, Han JX, Gao Y, Xie Y, Chen J, et al. Deficiency of BCAT2-mediated branched-chain amino acid catabolism promotes colorectal cancer development. Biochim Biophys Acta Mol Basis Dis. 2024;1870:166941. 37926361 10.1016/j.bbadis.2023.166941

[50] Ma Y, Li J, Wang H, Chiu Y, Kingsley CV, Fry D, et al. Combination of PD-1 inhibitor and OX40 agonist induces tumor rejection and immune memory in mouse models of pancreatic cancer. Gastroenterology. 2020;159:306–19.e12. 32179091 10.1053/j.gastro.2020.03.018 PMC7387152

[51] An W, Yang Q, Xi Y, Pan H, Huang H, Chen Q, et al. Identification of SRSF10 as a promising prognostic biomarker with functional significance among SRSFs for glioma. Life Sci. 2024;338:122392. 38160788 10.1016/j.lfs.2023.122392

[52] Luo X, Zhang Z, Li S, Wang Y, Sun M, Hu D, et al. SRSF10 facilitates HCC growth and metastasis by suppressing CD8(+)T cell infiltration and targeting SRSF10 enhances anti-PD-L1 therapy. Int Immunopharmacol. 2024;127:111376. 38113691 10.1016/j.intimp.2023.111376

[53] Li X, Yao W, Yuan Y, Chen P, Li B, Li J, et al. Targeting of tumour-infiltrating macrophages via CCL2/CCR2 signalling as a therapeutic strategy against hepatocellular carcinoma. Gut. 2015;66:157–67. 26452628 10.1136/gutjnl-2015-310514

[54] Zhou C, Weng J, Liu C, Liu S, Hu Z, Xie X, et al. Disruption of SLFN11 deficiency–induced CCL2 signaling and macrophage M2 polarization potentiates anti–PD-1 therapy efficacy in hepatocellular carcinoma. Gastroenterology. 2023;164:1261–78. 36863689 10.1053/j.gastro.2023.02.005

[55] Jia ZC, Das D, Zhang Y, Fernie AR, Liu YG, Chen M, et al. Plant serine/arginine-rich proteins: versatile players in RNA processing. Planta. 2023;257:109. 37145304 10.1007/s00425-023-04132-0

[56] Califice S, Baurain D, Hanikenne M, Motte P. A single ancient origin for prototypical serine/arginine-rich splicing factors. Plant Physiol. 2012;158:546–60. 22158759 10.1104/pp.111.189019 PMC3271749

[57] Fu XD. The superfamily of arginine/serine-rich splicing factors. Rna. 1995;1:663–80. 7585252 PMC1369309

[58] Jiang Y, Liu X, Fu J, Wu Y, Yu S, Yao K. Alternative splicing dysregulation in retinitis pigmentosa: pathogenic mechanisms and therapeutic opportunities. Biomolecules. 2025;15:1624. 41301541 10.3390/biom15111624 PMC12650716

[59] Lau C-K, Diem MD, Dreyfuss G, Van Duyne GD. Structure of the Y14-Magoh core of the exon junction complex. Curr Biol. 2003;13:933–41. 12781131 10.1016/s0960-9822(03)00328-2

[60] Das S, Krainer AR. Emerging functions of SRSF1, splicing factor and oncoprotein, in RNA metabolism and cancer. Mol Cancer Res. 2014;12:1195–204. 24807918 10.1158/1541-7786.MCR-14-0131 PMC4163531

[61] Bradley RK, Anczuków O. RNA splicing dysregulation and the hallmarks of cancer. Nat Rev Cancer. 2023;23:135–55. 36627445 10.1038/s41568-022-00541-7 PMC10132032

[62] Shkreta L, Delannoy A, Salvetti A, Chabot B. SRSF10: an atypical splicing regulator with critical roles in stress response, organ development, and viral replication. Rna. 2021;27:1302–17. 34315816 10.1261/rna.078879.121 PMC8522700

