# Supplementary material for: Disrupting SRSF10-dependent BCAT2 exon skipping reprograms tumor-associated macrophages and enhances anti-PD-1 efficacy in gastric cancer
Source: Cell Death Dis. 2026 Apr 22;17(1):536. doi: 10.1038/s41419-026-08622-3 (PMC13237086; doi:10.1038/s41419-026-08622-3)
Supplement: Supplementary file 1 — Supplemental methods, figures and tables [file 41419_2026_8622_MOESM1_ESM.pdf]

Supplemental Methods

Histology and immunostaining

Gastric tissues were collected and fixed overnight in 4% paraformaldehyde (Cat# P1110; Solarbio, Beiing, China), followed by paraffin embedding as previously described[1]. Sections (4 μm) were cut, stained with hematoxylin and eosin (H&E), and histologically verified by a board-certified pathologist. For immunohistochemistry (IHC), paraffin sections were deparaffinized in xylene and rehydrated through a graded ethanol series. Antigen retrieval was performed in sodium citrate buffer (MXB Biotechnology, Beijing, China), and endogenous peroxidase activity was quenched with hydrogen peroxide (MXB Biotechnology). Sections were then blocked in phosphate-buffered saline (PBS) containing 0.2% Triton X-100 and 5% bovine serum albumin (BSA, Cat# A8010, Solarbio) for 2 h at room temperature, followed by overnight incubation at 4 °C with primary antibodies. The following day, sections were incubated with HRP-conjugated secondary antibodies (Zhongshan Biotechnology, Guangdong, China) for 30 min at room temperature. Signals were visualized using diaminobenzidine (Zhongshan Biotechnology) and counterstained with hematoxylin. The antibodies used are listed in **Supplementary Table 1**. IHC scores (range 0–9) were calculated by multiplying intensity and heterogeneity scores as previously described[2, 3], and patients were classified into low (score 0, 1, 2, or 3) and high (score 4, 6, or 9) expression groups.

Immunofluorescence (IF) assays were performed as previously described[4, 5]. Briefly, sections were blocked in 5% BSA with 0.2% Triton X-100 for 1 h, followed by overnight incubation at 4 °C with primary antibodies. After washing, sections were incubated with fluorescence-conjugated secondary antibodies for 2 h at room temperature. Nuclei were counterstained with 4′,6-diamidino-2-phenylindole (DAPI, Cat# C0065, Solarbio). Images were acquired using a TCS SP8 confocal microscope (Leica Microsystems, Wetzlar, Germany).

Human samples

Between 2010 and 2015, patients diagnosed with GC at Fujian Medical University Union Hospital (FMUUH) had samples of their gastric cancer (GC), metaplastic, and normal gastric tissue collected. The inclusion criteria were: (a) histologically confirmed gastric cancer (GC); (b) no other malignancies; and (c) initial or updated tumour staging according to the 7th edition of the American Joint Committee on Cancer (AJCC) Cancer Staging Manual[6]. Tumor recurrence was defined as either biopsy-confirmed disease or imaging findings consistent with recurrence, and was classified based on anatomical location. Fresh intestinal-type GC tissue samples were also obtained from FMUUH patients for RNA sequencing (RNA-seq) and subsequent analysis of RRGs (RNA regulatory genes). This study was approved by the Ethics

Committee of FMUUH and written informed consent was obtained from all enrolled patients.

Bulk RNA-seq

Total mRNA was isolated and cut into short fragments from fresh frozen tissue. For cDNA synthesis, random hexamer-primer, buffer, dNTPs, RNase H and DNA polymerase I was used. After purifying with QIAQuick PCR extraction kit (Qiagen, Hilden, Germany), the cDNA was resolved with EB buffer for end-repair, A-base addition and ligation of the sequencing adapters. After that, suitable fragments were selected as templates for PCR amplification. Finally, paired-end libraries were sequenced using Illumina NovaSeq6000 platform, with a sequence coverage of 150bp paired-end reads.

By filtering out reads with low quality and removing sequencing adapters with fastp program, fastq files of 22 samples were mapped to the human reference genome hg19. And the Reads Per Kilobase of exon model per Million mapped fragments (RPKM) values were obtained using hisat2-RNASeQC2 pipeline.

Sample collection and processing for scRNA-seq

Eighteen gastric tissue samples were freshly obtained from laparoscopic surgeries of individuals with gastric cancer (GC) at Fujian Medical University Union Hospital (FMUUH). All freshly resected samples were divided into two parts: one processed for the scRNA-seq and the other embedded in paraffin. Histological verification of the normal gastric tissues was conducted by clinical pathologists using hematoxylin and eosin (H&E) staining.

The freshly collected tissue samples were washed with PBS upon saline. Tissue digestion was then performed by incubating the samples in a digestive enzyme mixture containing 1 mg/ml type IV collagenase (Sigma-Aldrich, St Lois, MO, USA), 10 ml RPMI-1640 (Gibco), 2 mg/ml dispase (Roche), and 10 U/μl DNase I (Roche) for 30 minutes at 37°C. The reaction was stopped by adding 10% fetal bovine serum (Gibco). Subsequently, the cell suspensions were filtered through a 70 μm filter and centrifuged at 500 rpm for 6 minutes at 4°C to pellet dead cells and red blood cells. The cells were washed twice with PBS and re-suspended in PBS supplemented with 0.5% BSA (Cat# A8010, Solarbio).

Library preparation and single-cell sequencing

Viable cells sorted by FACS were loaded into the well s of a microfluidic chip to prepare a cDNA library using a droplet-based sequencing platform (10x Genomics). Single-cell transcriptomic amplification and library preparation were conducted using the single-cell 3′ Library & Gel Bead Kit v2 (10x Genomics) following the manufacturer's instructions. The

libraries were sequenced using an Illumina sequencer (Illumina). Cell Ranger v3.1.0 (10x Genomics) was used with the 'mkfastq' and 'count' commands, applying default parameters and aligning to the GRCh38/hg38 reference genome. This process generated a matrix of unique molecular identifier (UMI) counts per gene along with the associated cell barcodes.

Quality control, cell clustering, and annotation

The Seurat package in R software (version 4.2.2) was used for analysis. Detailed quality control metrics are generated and evaluated. We mapped UMIs to genes and subsequently removed low quality cells. Cells were flagged as poor quality if they met any of the following thresholds: 1) the number of expressed genes was lower than 400 or higher than 5,000, 2) 20% or more of UMIs were mapped to mitochondrial genes, and 3) cells had more than 50,000 total molecules (nCount_RNA).

Data normalization was performed using Seurat's "NormalizeData" function with "LogNormalize" as the normalization method (scale.factor = 100,000). Variable genes were detected using the "FindVariableFeatures" function (nfeatures = 2,000). The "FindIntegrationAnchors" function was used to integrate all gastric tissue samples. The scaled gene expression data were projected onto Principal Components (PCs). 50 PCs were used for non-linear dimensionality reduction using Uniform Manifold Approximation and Projection (UMAP). The "FindClusters" function in the Seurat package was then utilized to conduct cell clustering analysis by embedding cells into a graph structure with a resolution parameter set to 0.6. This process resulted in the identification of 32 clusters. We annotated epithelial cells based on the expression of curated known cell markers, such as EPCAM, KRT8, and PLVAP, as shown in **Figure S1A**.

For a secondary cluster analysis of epithelial cell lineages from this aggregated data, clusters of interest were identified and subset using the "SubsetData" function. Detection of variable genes, scaling with UMI regression, PCA, clustering, and UMAP were repeated as described above. Clusters were annotated based on the expression of curated known cell markers, as shown in **Figure 1C.**

FindAllMarkers function and GSEA were used for acquiring DEGs and differential pathways. We used Cellchat[7] to screen enriched ligand and receptor pairs for cell-cell interaction analysis.

Spontaneous mouse GC mode

The spontaneous gastric cancer (GC) model was established as previously described[8]. Briefly, wild-type C57BL/6 mice were administered 240 ppm N-methyl-N-nitrosourea (MNU; HY-

34758, MCE, USA) in their drinking water for 6 weeks, following a schedule of 1 week of exposure alternated with 1 week of withdrawal. At 36 weeks of age, mice were euthanized, and stomach tissues were harvested for subsequent analyses.

Tamoxifen administration

Mice were given 5 mg tamoxifen by subcutaneous injection at 8 weeks of age for 3 consecutive days as previously described[3, 9]. SPEM was induced by daily intraperitoneal injections of tamoxifen dissolved in 90% corn oil and 10% ethanol (5 mg/20 g body weight; T832955, Macklin, Inc., Rochelle, IL, USA).

Cell culture, transfection, and lentivirus infection

The human gastric carcinoma (GC) cell line (AGS) was obtained from Cellcook Biotechnology Ltd (Guangzhou, China). All cell lines used in this study were confirmed to be mycoplasma-free and characterised using a short tandem repeat (STR) profile. The cells were grown in F-12K medium (Gibco, Waltham, MA, USA), supplemented with 10% fetal bovine serum (Gibco) and penicillin/streptomycin (Cat# BL505A, Biosharp). All cell lines were cultured in a 5% CO_2 incubator at 37 °C. Transient transfection of plasmids was performed using Lipofectamine 3000 reagent (Thermo Fisher Scientific) according to the manufacturer's instructions. To generate stable cell lines, cells were infected with lentivirus for 12–24 hours at a low fusion level (20%). After 72 hours, the cells were screened with puromycin (Sigma-Aldrich) for two weeks.

Cell proliferation and cell cycle assays

Cell proliferation was assessed using the Cell Counting Kit-8 (CCK-8; Dojindo, Kumamoto, Japan) according to the manufacturer's instructions[10]. Briefly, approximately 2×10^3 cells were seeded into 96-well plates, after which 10 μL of CCK-8 reagent was added to each well, and cells were incubated for the indicated time periods. Optical density (OD) at 450 nm was measured using a microplate reader (Bio-Rad, Hercules, CA, USA).

For cell cycle analysis, approximately 5×10^5 cells were seeded into 6-cm dishes and cultured for 24 h. Cells were then harvested, fixed in 70% ethanol at 4 °C overnight, and washed with PBS. Fixed cells were stained with propidium iodide (PI) and filtered through a 70-μm cell strainer before analysis on a FACSVerse flow cytometer (BD Biosciences, Franklin Lakes, NJ, USA).

RNA stability assay

AGS cells were seeded into 6-well plates to get 50% confluency after 24h. Cells were treated

with 5 ug/ml actinomycin D (SBR00013, Sigma) and collected at indicated time points. Total RNA was extracted with TRIzol and analysed by RT–PCR. GAPDH served as the internal reference, and the data were analyzed using the 2−ΔΔCT method.

Gastric cancer xenograft models

A total of 2×10^6 stably transduced AGS cells suspended in 0.1 mL PBS were subcutaneously injected into the right flank of C57BL/6 mice. Five mice were used in each experimental group. After 25 days, mice were euthanized, and subcutaneous tumors were excised. Tumor volumes were measured with calipers and calculated using the formula: $V = (L \times W^2)/2$ (V, tumor volume; L, length; W, width).

Western blotting

Cells were lysed in RIPA buffer (Beyotime, Shanghai, China) supplemented with a protease and phosphatase inhibitor cocktail (Sigma-Aldrich, St. Louis, MO, USA). Protein concentrations were determined using a BCA Protein Assay Kit (Thermo Scientific, Waltham, MA, USA). Equal amounts of protein (30–80 μg) were separated by SDS-PAGE and transferred onto polyvinylidene difluoride (PVDF) membranes (Millipore, Burlington, MA, USA). Membranes were blocked with 5% non-fat dry milk in TBST for 2 h at room temperature, followed by overnight incubation at 4 °C with primary antibodies. After washing, membranes were incubated with appropriate HRP-conjugated secondary antibodies, and signals were visualized using an ImageQuant LAS 4000 Mini system (GE Healthcare, Chicago, IL, USA).

Quantitative real-time PCR

Total RNA was extracted using TRIzol reagent (Invitrogen, Carlsbad, CA, USA), and 800 ng of RNA was reverse transcribed into cDNA using PrimeScript RT Master Mix (Takara, Shiga, Japan) according to the manufacturer's instructions. Quantitative real-time PCR was performed using SYBR Green PCR Master Mix (Takara) on a real-time PCR system (Bio-Rad, Hercules, CA, USA). Relative gene expression was calculated using the $2^{-\Delta\Delta CT}$ method[11]. The primer sequences used in this study are listed in **Supplementary Table 2**.

RNA immunoprecipitation

RIP was performed per the manufacturer's protocol using a Magna MeRIP Kit (17-10499, Merck Millipore, USA), and the immunoprecipitated RNA was analyzed using qRT-PCR.

Analysis of publicly available datasets

Public RNA-seq data for stomach adenocarcinoma (TCGA-STAD) were queried via GEPIA2 (http://gepia2.cancer-pku.cn; accessed on November 17, 2025). GEPIA2 uses the UCSC Xena/Toil harmonized pipeline. Expression values are TPM transformed and visualized as $\log_2(TPM+1)$. The specific operational procedures are outlined in the report[12].

References

1. Hagen SJ, Ang LH, Zheng Y, Karahan SN, Wu J, Wang YE, et al. Loss of Tight Junction Protein Claudin 18 Promotes Progressive Neoplasia Development in Mouse Stomach. Gastroenterology. 2018;155(6):1852-67.
2. Xie JW, Huang XB, Chen QY, Ma YB, Zhao YJ, Liu LC, et al. m(6)A modification-mediated BATF2 acts as a tumor suppressor in gastric cancer through inhibition of ERK signaling. Mol Cancer. 2020;19(1):114.
3. Huang XB, Huang Q, Jiang MC, Zhong Q, Zheng HL, Wang JB, et al. KLHL21 suppresses gastric tumourigenesis via maintaining STAT3 signalling equilibrium in stomach homoeostasis. Gut. 2024;73(11):1785-98.
4. Bockerstett KA, Lewis SA, Noto CN, Ford EL, Saenz JB, Jackson NM, et al. Single-Cell Transcriptional Analyses Identify Lineage-Specific Epithelial Responses to Inflammation and Metaplastic Development in the Gastric Corpus. Gastroenterology. 2020;159(6):2116-29.e4.
5. Schwarz P, Kübler JA, Strnad P, Müller K, Barth TF, Gerloff A, et al. Hepcidin is localised in gastric parietal cells, regulates acid secretion and is induced by Helicobacter pylori infection. Gut. 2012;61(2):193-201.
6. Rice TW, Blackstone EH, Rusch VW. 7th edition of the AJCC Cancer Staging Manual: esophagus and esophagogastric junction. Ann Surg Oncol. 2010;17(7):1721-4.
7. Jin S, Guerrero-Juarez CF, Zhang L, Chang I, Ramos R, Kuan C-H, et al. Inference and analysis of cell-cell communication using CellChat. Nat Commun. 2021;12(1):1088.
8. Miao ZF, Sun JX, Adkins-Threats M, Pang MJ, Zhao JH, Wang X, et al. DDIT4 Licenses Only Healthy Cells to Proliferate During Injury-induced Metaplasia. Gastroenterology. 2021;160(1):260-71.e10.
9. Radyk MD, Burclaff J, Willet SG, Mills JC. Metaplastic Cells in the Stomach Arise, Independently of Stem Cells, via Dedifferentiation or Transdifferentiation of Chief Cells. Gastroenterology. 2018;154(4):839-43.e2.
10. Chen QY, Huang XB, Zhao YJ, Wang HG, Wang JB, Liu LC, et al. The peroxisome proliferator-activated receptor agonist rosiglitazone specifically represses tumour metastatic potential in chromatin inaccessibility-mediated FABP4-deficient gastric cancer. Theranostics. 2022;12(4):1904-20.
11. Livak KJ, Schmittgen TD. Analysis of relative gene expression data using real-time quantitative PCR and the 2(-Delta Delta C(T)) Method. Methods. 2001;25(4):402-8.
12. Tang Z, Li C, Kang B, Gao G, Li C, Zhang Z. GEPIA: a web server for cancer and normal gene expression profiling and interactive analyses. Nucleic Acids Research. 2017;45(W1).

Supplementary figures

Figure S1

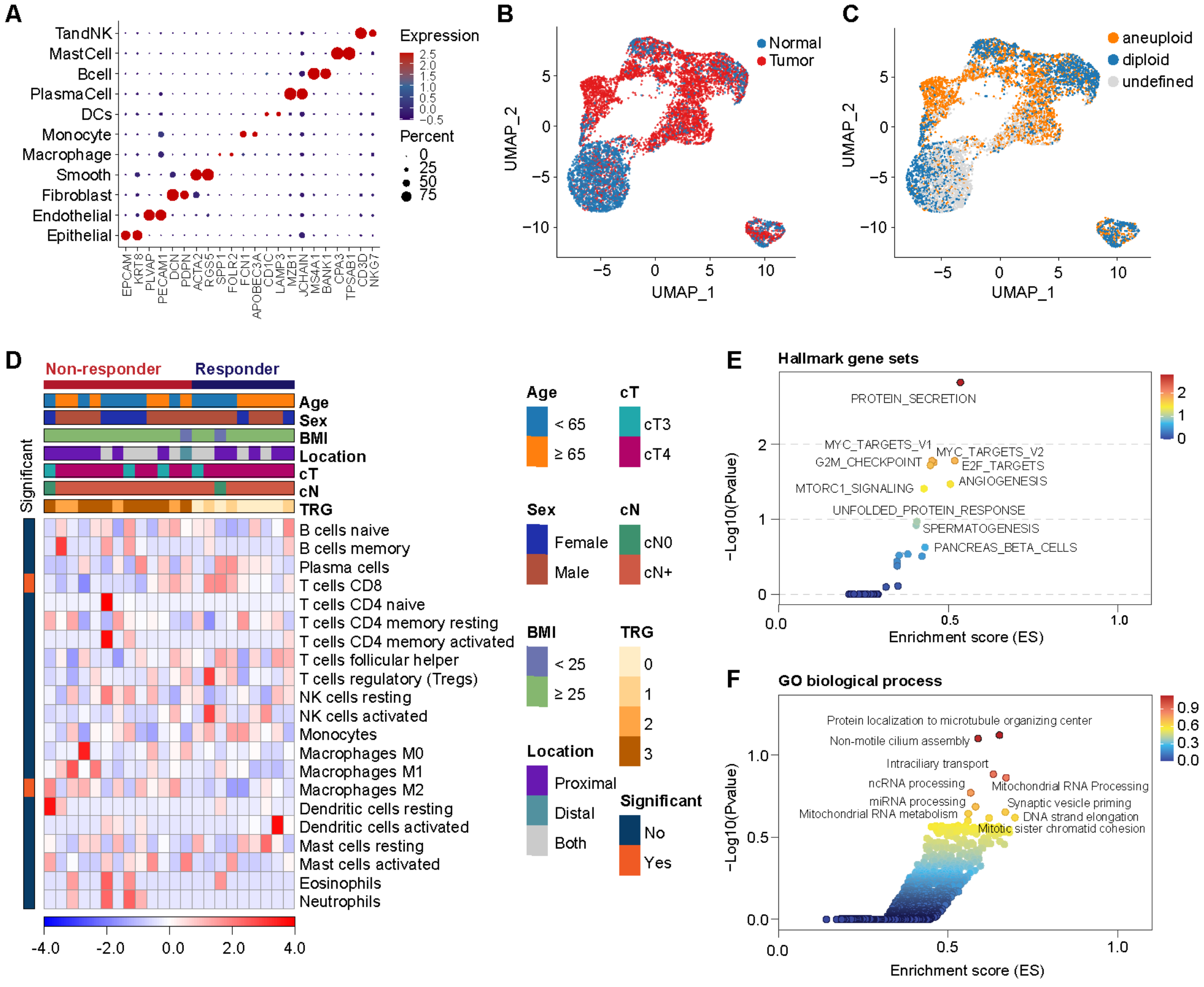

Figure S1. Integrated single-cell and bulk transcriptomic analyses of gastric cancer. A. Dot plot showing canonical marker genes used to define major gastric cell populations. **B-C.** UMAP plots illustrating the distribution of epithelial cells by sample origin (Normal vs Tumor) and ploidy status (aneuploid, diploid, undefined). **D.** Immune landscape comparison between responders and non- responders to ICIs. Upper panels show clinical characteristics (age, sex, BMI, tumor location, clinical T/N stage, TRG). Lower heatmap shows differential immune infiltration estimated by CIBERSORT. **E.** Hallmark gene set enrichment analysis of bulk RNA-seq (non-responders vs. responders) showing enrichment of pathways related to protein secretion, MYC targets, G2M checkpoint, and mTORC1 signaling. **F.** GO biological process enrichment highlighting RNA processing, mitochondrial RNA metabolism, and cell cycle–related pathways.

Figure S2

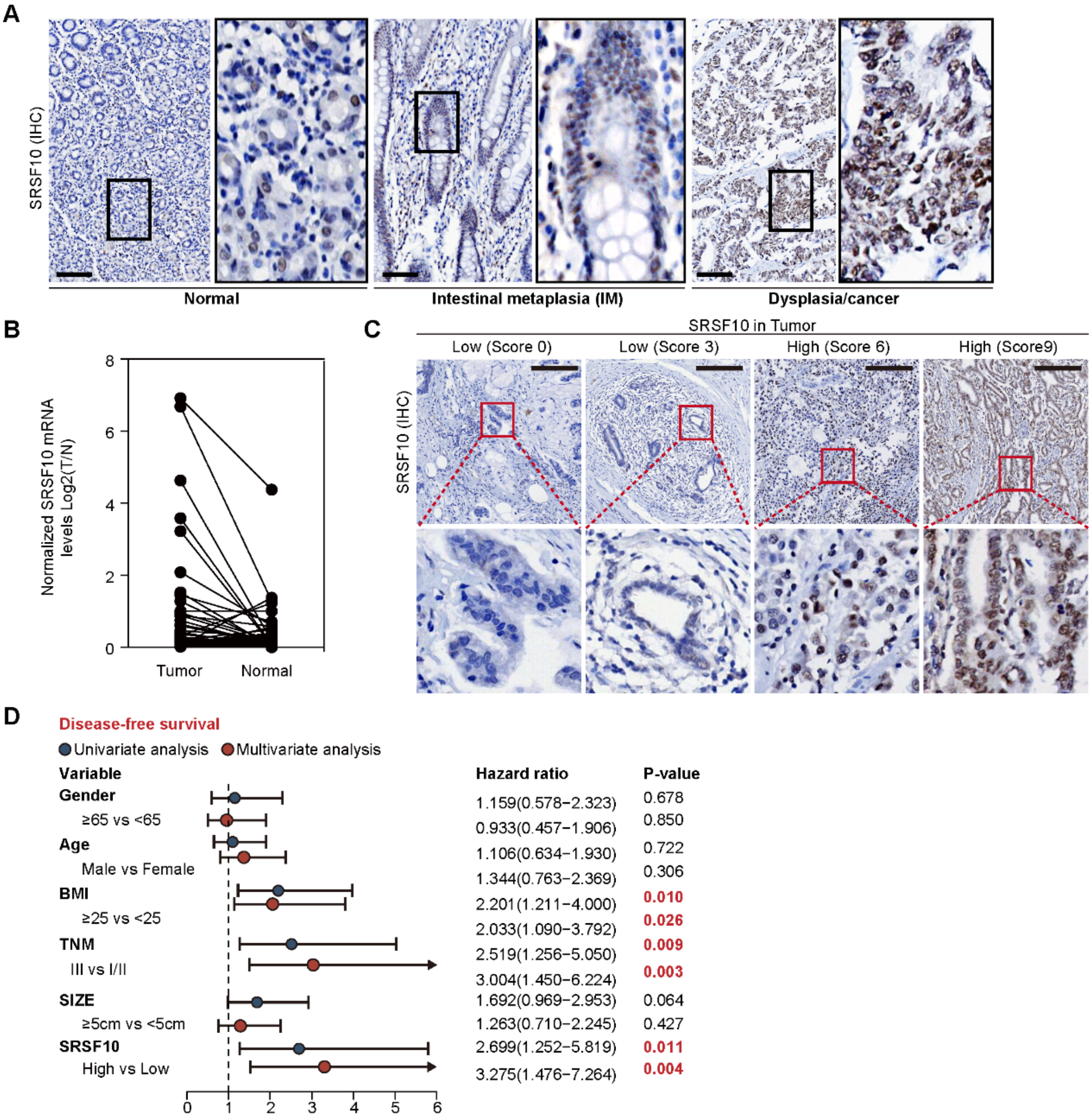

Figure S2. Validation of SRSF10 expression and its prognostic significance in gastric cancer. A. IHC images of SRSF10 in different gastric tissue states (normal, intestinal metaplasia, dysplasia/cancer). **B.** qPCR validation of SRSF10 expression in paired gastric tumor and adjacent normal tissues. **C.** IHC scoring of SRSF10 expression in gastric tumors, illustrating representative cases with low (score 0, 3) and high expression (score 6, 9). **D.** Univariate and multivariate Cox regression analyses for disease-free survival (DFS). Scale bars in **A** and **C** ,100 μm.

Figure S3

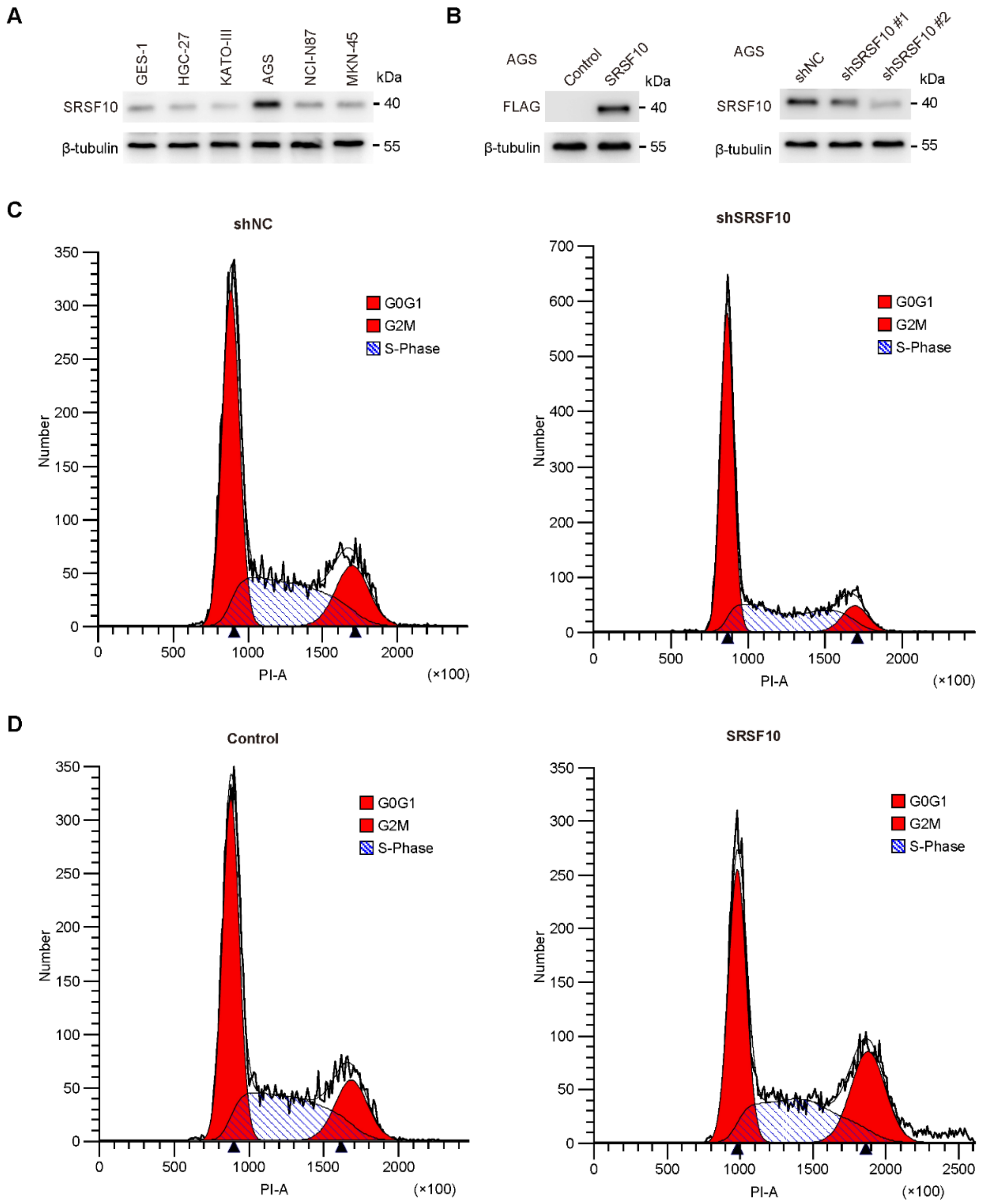

Figure S3. SRSF10 knockdown and overexpression validated in GC cell lines. A. Western blot analysis of SRSF10 protein expression in various gastric cancer cell lines (HGC-27, KATO-III, AGS, NCI-N87, MKN-45) and a normal gastric epithelial cell line (GES-1). **B.** Western blot analysis of FLAG in AGS cells transfected with a control vector or an SRSF10 expression vector (left). Western blot analysis of SRSF10 in AGS cells transfected with shNC, shSRSF10 #1, or shSRSF10 #2(right). **C.** Flow cytometry analysis of cell cycle distribution in AGS cells transfected with shNC or shSRSF10. **D.** Flow cytometry analysis of cell cycle distribution in AGS cells transfected with a control vector or an SRSF10 expression vector.

Figure S4

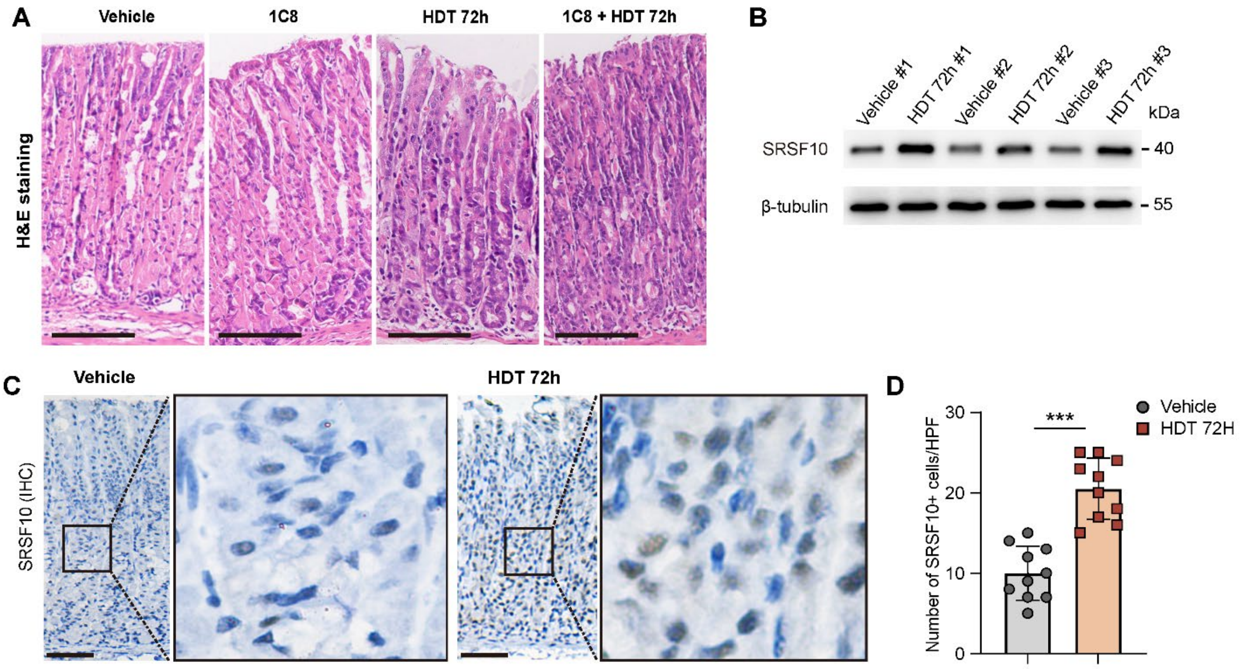

FigureS4. HDT induces spasmolytic polypeptide-expressing metaplasia (SPEM). A. Representative H&E staining of gastric corpus tissues from mice treated with vehicle, 1C8, high-dose tamoxifen (HDT, 72 h), or combination (1C8+ HDT). **B.** Western blot of SRSF10 in whole-stomach lysates from vehicle and HDT 72 h mice. **C.** Representative SRSF10 IHC images from vehicle and HDT 72 h stomachs. **D.** Quantification of SRSF10^{+} epithelial cells per high-power field (HPF). Scale bars in **A** and **C** ,100 μm.

Figure S5

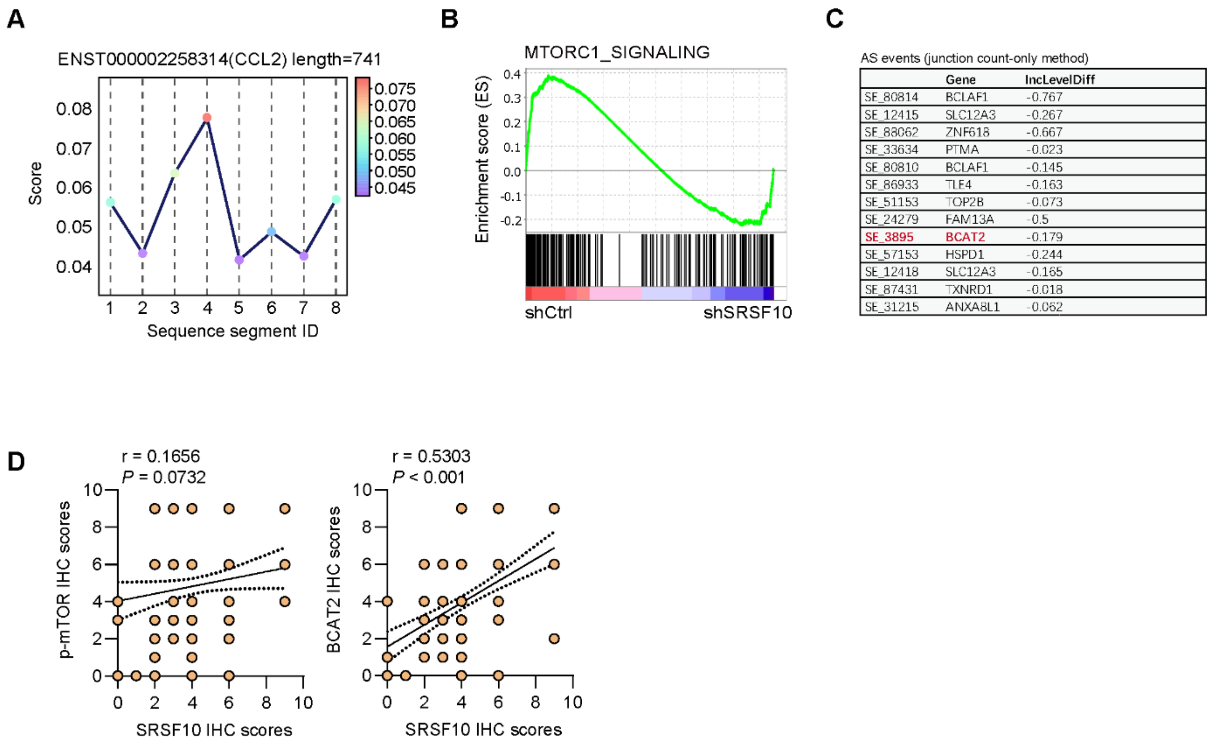

	Gene	IncLevelDiff
SE_80814	BCLAF1	-0.767
SE_12415	SLC12A3	-0.267
SE_88062	ZNF618	-0.667
SE_33634	PTMA	-0.023
SE_80810	BCLAF1	-0.145
SE_86933	TLE4	-0.163
SE_51153	TOP2B	-0.073
SE_24279	FAM13A	-0.5
SE_3895	BCAT2	-0.179
SE_57153	HSPD1	-0.244
SE_12418	SLC12A3	-0.165
SE_87431	TXNRD1	-0.018
SE_31215	ANXA8L1	-0.062

Figure S5. SRSF10 regulates BCAT2 splicing and correlates with BCAT2 in GC tissues. **A.** Schematic of the CCL2 mRNA transcript (E NST000002258314) structure with sequence segment scores indicating potential SRSF10 binding sites across 8 segments. **B.** Gene set enrichment analysis (GS EA) of mTORC1_SIGNALING in gastric cancer cells transfected with shCtrl or shSRSF10. **C.** Table of significantly altered alternative splicing (AS) events detected using the junction count-only method. **D.** Correlation analyses of SRS F10 IHC scores with p-mTOR (left) and BCAT2 (right) IHC scores in gastric cancer tissues.

Figure S6

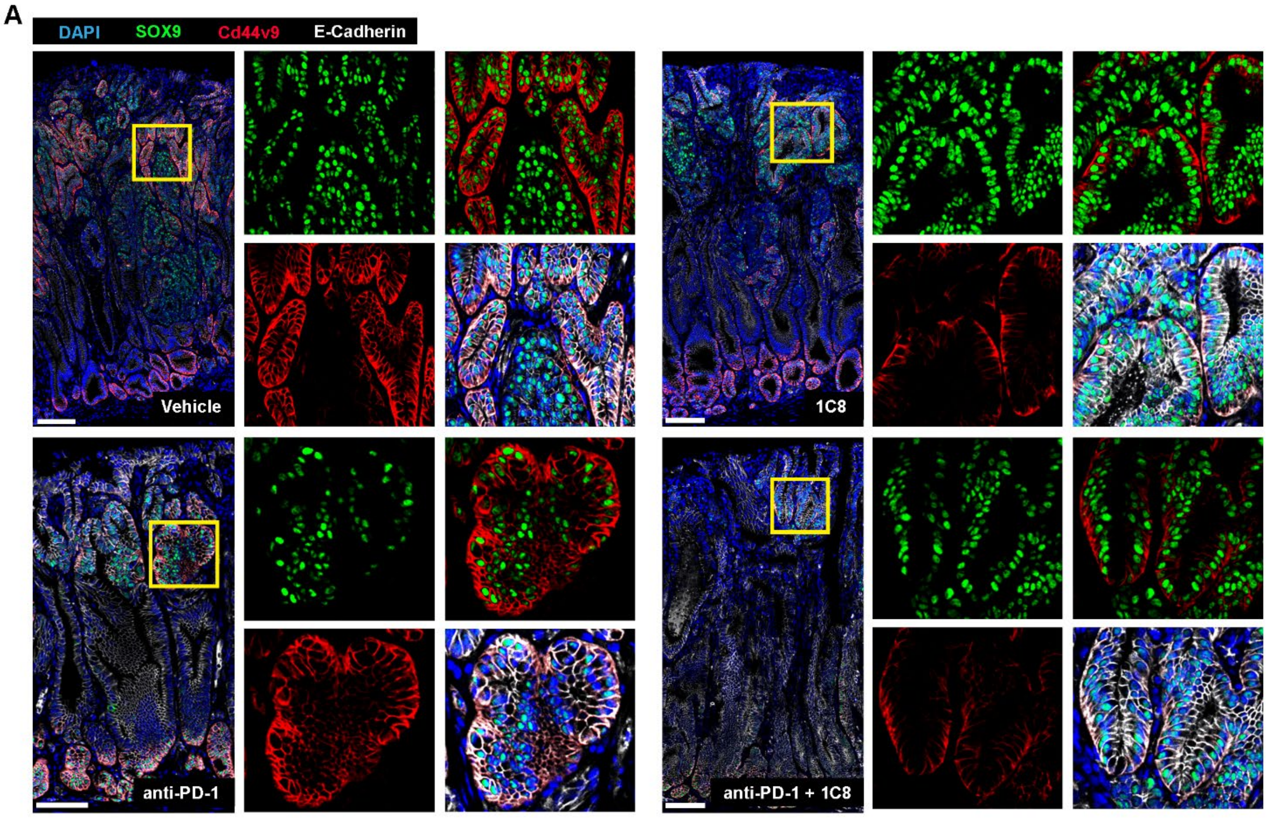

Figure S6. Combined 1C8 and anti–PD-1 suppress metaplastic cell populations. A. Immunofluorescence staining images of gastric tissue from mice in different treatment groups (vehicle, 1C8, anti-PD-1 and anti-PD-1+1C8). SOX9 (green), CD44v9 (red), E-Cadherin(white) and DAPI (blue). Scale bars in **A**,100 μm.

Supplementary Tables.

**Supplementary Table 1. Antibodies application and dilution.**			
**Antibodies**	**Company**	**Application**	**Dilution**
SRSF10	Abcam (ab254935)	IHC,WB	1:400 (IHC), 1:2000 (WB)
CCL2	Abcam (ab315478)	IF,WB	1:100 (IF), 1:1000 (WB)
CD86	Abcam (ab220188)	IHC	1:1000 (IHC)
CD206	Abcam (ab220189)	IHC	1:1000 (IHC)
CD8	Abcam (ab217344)	IF,IHC	1:500 (IF), 1:2000 (IHC)
GzmB	Abcam (ab255598)	IF	1:200 (IF)
GIF	Santa Cruz (sc-514524)	IF	1:200 (IF)
GS-II	Invitrogen (L32451)	IF	1:200 (IF)
TROP2	Invitrogen (PA5-47074)	IF	1:100 (IF)
tdTomato	Bioryt (orb182397)	IF	1:100 (IF)
CD44v9	Cosmo Bio (LKG-M002)	IF	1:2000 (IF)
E-Cadherin	Huabio (EM0502)	IF	1:200 (IF)
Ki67	Invitrogen (14-5698-82)	IF	1:200 (IF)
DYKDDDDK	Cell Signaling (#8146S)	WB	1:1000 (WB)
β-tubulin	Proteintech (10094-1-AP)	WB	1:2000 (WB)
p-mTOR	Proteintech (67778-1-Ig)	IHC,WB	1:500 (IHC), 1:1000 (WB)
SOX9	Millipore (Ab5535)	WB,IF	1:500 (WB), 1:400 (IF)
CD44	Abcam (ab157107)	WB	1:2000 (WB)
BCAT2	Proteintech (16417-1-AP)	IHC,WB	1:200 (IHC), 1:1000 (WB)

Supplementary Table 2. List of primers used for qPCR.		
**Gene**	**Primer**	**Sequence**
CA9(homo)	Forward	GACTTCAGCCGCTACTTCCA
CA9(homo)	Reverse	TCAGCTGTAGCCGAGAGTCA
HSPA1A(homo)	Forward	AGCTGGAGCAGGTGTGTAAC
HSPA1A(homo)	Reverse	ACAGCAATCTTGGAAAGGCC
EGLN3(homo)	Forward	CGCTGCATCACCTGCATCTA
EGLN3(homo)	Reverse	ACTTCGTGTGGGTTCCTACG
HSPA1B(homo)	Forward	CCTACCATTGAGGAGGTGGATTA
HSPA1B(homo)	Reverse	TACAAAGAAGTGAAGCAGCAAAGA
PTPRQ(homo)	Forward	GCTGGCTATGGCAATGCTTC
PTPRQ(homo)	Reverse	CAGCAGGTTCACTCCAGCTT
MYO1A(homo)	Forward	TGGTGACTACATTGGGCTGC
MYO1A(homo）	Reverse	TGGTCAGGAGGAGAATCCGA
CCL2(homo）	Forward	CAGCAAGTGTCCCAAAGAAGC
CCL2(homo)	Reverse	TCGGAGTTTGGGTTTGCTTG
TGM3(homo)	Forward	ATGGCAGGTGTTGGATGCTACC
TGM3(homo)	Reverse	CCGCGAAGATAAAGGGCATGTC
CP(homo）	Forward	TTCACGGCCATAGCTTCCAAT
CP(homo）	Reverse	CAGCATGAATGTGGTCGGTC
TNF(homo）	Forward	TCCTCTCTGCCATCAAGAGC
TNF(homo）	Reverse	AGTAGACCTGCCCAGACTCG
NOS2(homo）	Forward	TCACCTACTTCCTGGACATCAC
NOS2(homo）	Reverse	GAACTTCCACTTGCTGTACTCTG
CXCL9(homo）	Forward	CCTCTTGGGCATCATCTTGCT

CXCL9(homo)	Reverse	GATAGTCCCTTGGTTGGTGCT
CXCL10(homo)	Forward	GTGGCATTCAAGGAGTACCTC
CXCL10(homo)	Reverse	TGATGGCCTTCGATTCTGGATT
CD206(homo)	Forward	TGGTGAACGGAATGATTGTGTAG
CD206(homo)	Reverse	GGTCCATCTTCCTTGTGTCAG
CD163(homo)	Forward	GGACCGATATGGCTCAATGAAG
CD163(homo)	Reverse	TAAAGGATGACTGACGGGATGA
TGF-β1(homo)	Forward	GAGCCCTGGACACCAACTAT
TGF-β1(homo)	Reverse	AAGTTGGCATGGTAGCCCTT
SRSF10(homo)	Forward	TTCTACACTCGCCGTCCAAG
SRSF10(homo)	Reverse	TTCAATCTGCCGTCCACAAATC
