# Supplementary figures and images for: Disrupting SRSF10-dependent BCAT2 exon skipping reprograms tumor-associated macrophages and enhances anti-PD-1 efficacy in gastric cancer
Source: Cell Death Dis. 2026 Apr 22;17(1):536. doi: 10.1038/s41419-026-08622-3 (PMC13237086; doi:10.1038/s41419-026-08622-3)

Full length western blots

Figure 3C

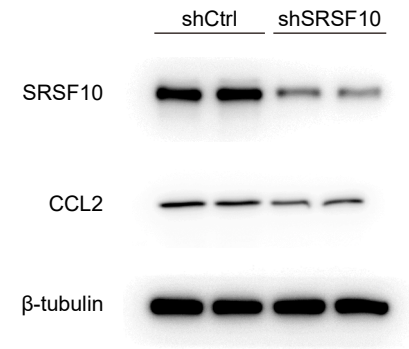

Figure 5C

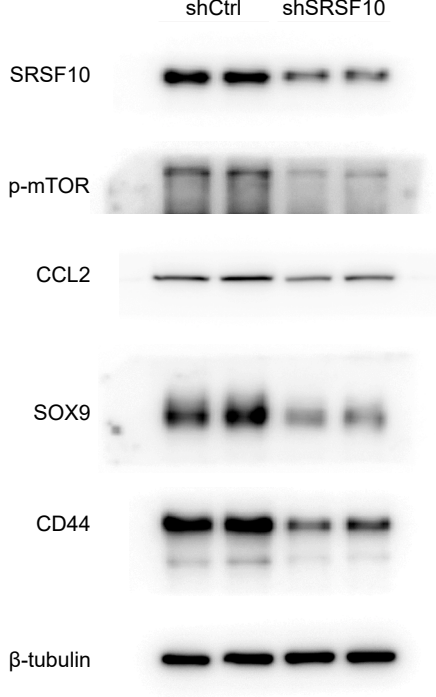

Figure S3 A

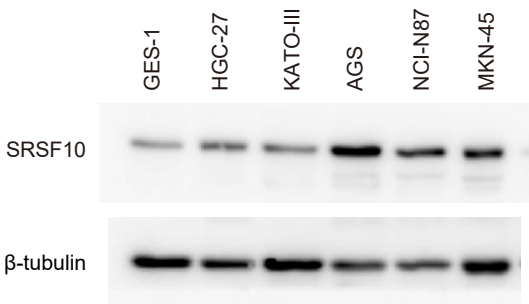

Figure 5K

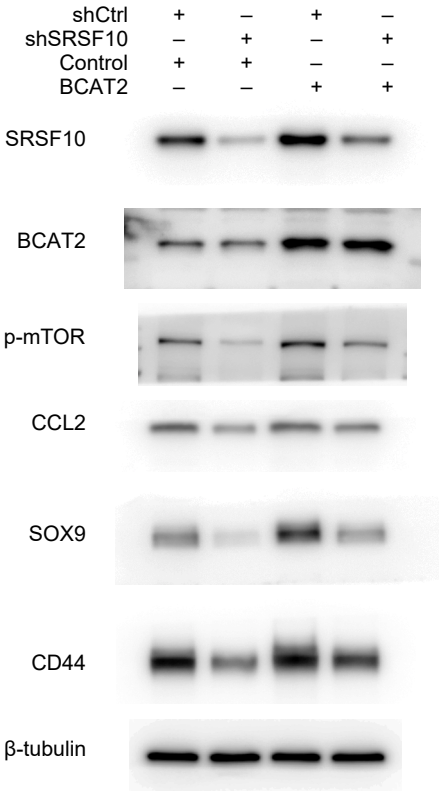

Figure S3 B

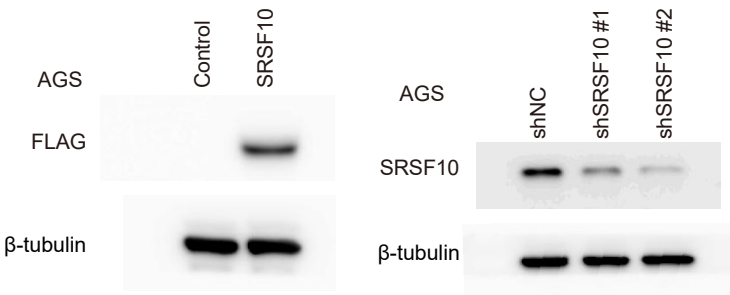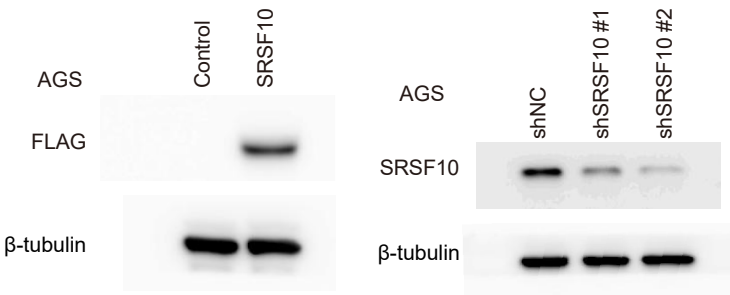

Supplement: Supplementary file 2 — Full length western blots [file 41419_2026_8622_MOESM2_ESM.pdf]
